# Self-Assembled NBR/Nomex Nanofibers as Lightweight Rubbery Nonwovens for Hindering Delamination in Epoxy CFRPs

**DOI:** 10.1021/acsami.1c17643

**Published:** 2021-12-23

**Authors:** Emanuele Maccaferri, Laura Mazzocchetti, Tiziana Benelli, Tommaso Maria Brugo, Andrea Zucchelli, Loris Giorgini

**Affiliations:** † Department of Industrial Chemistry “Toso Montanari”, University of Bologna, Viale Risorgimento 4, 40136 Bologna, Italy; ‡ Interdepartmental Center for Industrial Research on Advanced Applications in Mechanical Engineering and Materials Technology, CIRI-MAM, University of Bologna, Viale Risorgimento 2, 40136 Bologna, Italy; § Department of Industrial Engineering, University of Bologna, Viale Risorgimento 2, 40136 Bologna, Italy

**Keywords:** nitrile butadiene rubber, Nomex, electrospinning, mixed nanofiber, CFRP, rubber, delamination, toughening

## Abstract

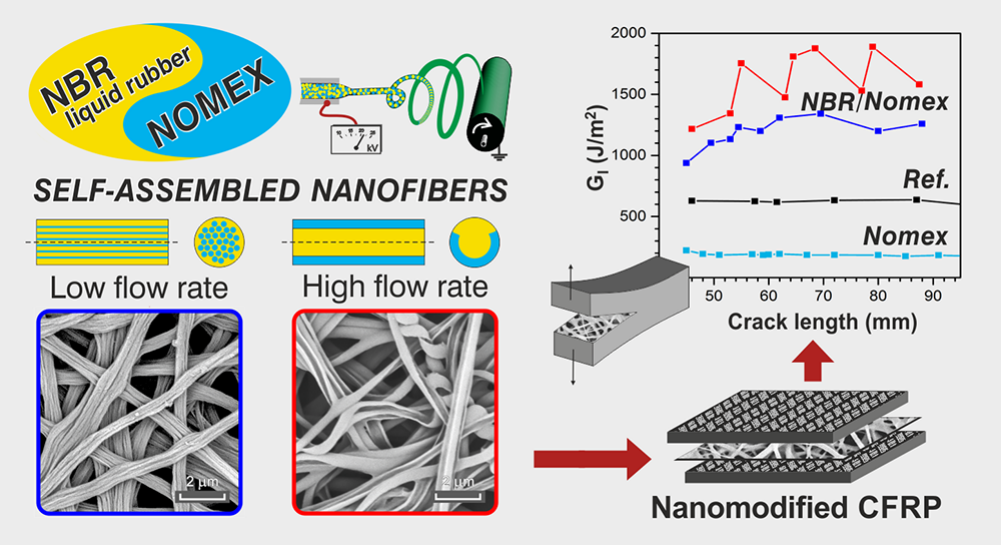

Still today, concerns regarding delamination limit the widespread use of high-performance composite laminates, such as carbon fiber-reinforced polymers (CFRPs), to replace metals. Nanofibrous mat interleaving is a well-established approach to reduce delamination. However, nanomodifications may strongly affect other laminate thermomechanical properties, especially if achieved by integrating soft materials. Here, this limitation is entirely avoided by using rubbery nitrile butadiene rubber (NBR)/Nomex mixed nanofibers: neither laminate stiffness nor glass-transition temperature (*T*_g_) lowering occurs upon CFRP nanomodification. Stable noncrosslinked nanofibers with up to 60% wt of NBR were produced via single-needle electrospinning, which were then morphologically, thermally, spectroscopically, and mechanically characterized. NBR and Nomex disposition in the nanofiber was investigated via selective removal of the sole rubber fraction, revealing the formation of particular self-assembled structures resembling quasi-core–shell nanofibers or fibril-like hierarchical structures, depending on the applied electrospinning conditions (1.10 and 0.20 mL/h, respectively). Mode I and Mode II loading tests show a significant improvement of the interlaminar fracture toughness of rubbery nanofiber-modified CFRPs, especially *G*_I_ (up to +180%), while *G*_II_ enhancement is less pronounced but still significant (+40% in the best case). The two nanofibrous morphologies (quasi-core–shell and fibril-like ones) improve the delamination resistance differently, also suggesting that the way the rubber is located in the nanofibers plays a role in the toughening action. The quasi-core–shell nanofiber morphology provides the best reinforcing action, besides the highest productivity. By contrast, pure Nomex nanofibers dramatically worsen the interlaminar fracture toughness (up to −70% in *G*_I_), acting as a release film. The achieved delamination resistance improvements, combined with the retention of both the original laminate stiffness and *T*_g_, pave the way to the extensive and reliable application of NBR/Nomex rubbery nanofibrous mats in composite laminates.

## 1 Introduction

Searching for systems able to limit the catastrophic consequences of composite laminate failure is of primary importance for safety and economic reasons. Today, laminated fiber-reinforced polymers (FRPs), especially carbon FRPs (CFRPs), have widespread usage where lightweight and excellent specific stiffness and strength are required. Delamination, that is, the debonding of the constituent laminae due to the formation and propagation of microcracks, is the main failure mode of laminated structures.^1^ To avoid or, at least, limit this detrimental phenomenon and its catastrophic consequences, it is possible to act on two sides: (i) providing technical solutions for detecting hazardous conditions promptly and (ii) increasing the intrinsic delamination resistance of the laminate. In the first case, systems that allow structural health monitoring, like Bragg fibers or piezoelectric fibers, are exploited.^2,3^ The other way (case ii) aims at producing laminates with improved interlaminar fracture toughness, making the crack triggering more difficult and, in turn, the failure by delamination. Several solutions can be implemented, such as rubber addition. The rubber can be directly blended with the resin either as a “liquid”, that is, noncrosslinked elastomeric precursors, as crosslinked particles or as core–shell particles consisting of inner “liquid” rubber confined in an outer “rigid” thermoplastic shell.^4−8^ These approaches involve the modification of the resin bulk with a relatively high amount of toughener (5–20% wt) that potentially affects the outstanding elastic modulus, strength, and glass-transition temperature (*T*_g_) of the overall laminate.^6^ An alternative is the interleaving of rubber films, like Kraibon, for increasing composite damping or to reduce failures at the interface of dissimilar materials.^9^ While these films may improve the delamination performance, they strongly impact laminate dimensions and mechanical properties.^10^ Moreover, they are not lightweight since the rubber layers have sub-millimeter thickness.

To bypass these limitations, nanoreinforcements such as carbon nanotubes, carbon nanofibers, and polymeric nanofibers have been proposed as epoxy modifiers for increasing interlaminar fracture toughness.^11−17^ In particular, electrospun nonwoven layers are very promising: their integration among composite laminae may significantly increase the energy release rate (*G*_I_ and *G*_II_),^18^ provided that good adhesion between nanofibers and the matrix is established. The integration of nanofibrous layers has relevant advantages with respect to toughening of bulk resin. Indeed, nanomodified laminates can be engineered to attain tailored properties by choosing a suitable polymer besides the nanofibrous mat morphology, grammage, and the number and extent of reinforced interfaces. Thus, nanofibrous mats allow localized laminate modifications in areas where interlaminar stresses are mostly concentrated, like free edges, holes, ply-drops, and adhesive bondings,^19^ with a limited worsening of the laminate characteristics, such as elastic modulus, strength, *T*_g_, overall dimensions, and weight. However, almost all the proposed nanofibrous nonwovens are made by thermoplastic polymers, especially polyamides and polyesters,^18^ hindering delamination through the so-called “bridging” mechanism or by matrix toughening.^15^ In the first case, the three-dimensional nanofibrous net helps to keep adjacent laminae together, hampering the delamination. In the latter, the polymer mixes with the resin, making it less fragile by plasticizing it. In both cases, the energy required for the crack propagation increases. Amorphous low-*T*_g_ polymers, like rubbers, are useful to improve the interlaminar fracture toughness, as mentioned above. Unfortunately, shaping them into nanofibers is prevented by the rubber cold flow that leads to the formation of bulk films.

Recently, the authors proposed a simple method to produce stable rubbery nanofibers via single-needle electrospinning without the need for additional steps, like crosslinking.^20^ It was demonstrated that a semi-crystalline thermoplastic polymer with suitable characteristics (low *T*_g_ and the melting temperature, *T*_m_, above room temperature) blended with the rubber may produce a dimensionally stable nanofibrous morphology. The obtained structure, that is, nitrile butadiene rubber (NBR) blended with poly(ε-caprolactone) (PCL), behaves similar to a thermoplastic elastomer (TPE). The interleaving of such nonwovens in epoxy CFRP laminates provides an extraordinary enhancement of the interlaminar fracture toughness in Mode I (*G*_I_ up to +480%) and a slight improvement in Mode II, besides a better damping behavior.^21−23^ However, the resulting laminates suffer from some important *T*_g_ lowering, strongly depending on the nanomodification extent and the nanofiber rubber amount. Such results suggest the need for a compromise between improved interlaminar fracture toughness, damping, and overall mechanical properties. NBR/PCL blend nanofibers act exclusively as matrix toughening since the crystalline PCL fraction melts (*T*_m_ ≈ 60 °C) and the polymer pair mixes with the epoxy resin during the curing cycle. However, the combination of matrix toughening and nanofiber bridging mechanisms may work even better than single mechanisms individually taken.

Recent works^24,25^ investigated the role of PCL, deriving from the shell of coaxial nylon/PCL nanofibers, on the interlaminar fracture toughness. The so-called PCL “interdiffusion” into epoxy, that is, the polymer mixing with the hosting resin, has a relevant role in the final composite performance. In particular, when the curing temperature enables the PCL melting, the recorded interlaminar fracture toughness is higher than laminates cured at temperatures that prevent the interdiffusion of the low-*T*_m_ thermoplastic component.

Using NBR blended with a polymeric counterpart that does not melt or mixes with the matrix during the curing cycle could enable both the abovementioned toughening mechanisms.

Thus, in the present work, NBR solutions blended with Nomex, a polyaramid with high thermal properties, were electrospun via single-needle electrospinning. Dimensionally stable NBR/Nomex mixed nanofibers with up to 60% wt of rubber were produced and then they were morphologically, thermally, spectroscopically, and mechanically characterized before integrating into epoxy CFRP laminates. Morphological investigations reveal the formation of peculiar self-assembled structures that resemble quasi-core–shell nanofibers or fibril-like hierarchical structures, depending on the applied electrospinning conditions. The overall thermomechanical properties of laminates were assessed via dynamic mechanical analysis (DMA). Finally, the ability of NBR/Nomex rubbery nonwovens at contrasting delamination under Mode I and Mode II loadings [double cantilever beam, (DCB) and end-notched flexure (ENF) tests, respectively] was investigated in order to assess whether the two diverse detected morphologies provide different interlaminar fracture toughness effects.

**Figure 1:**
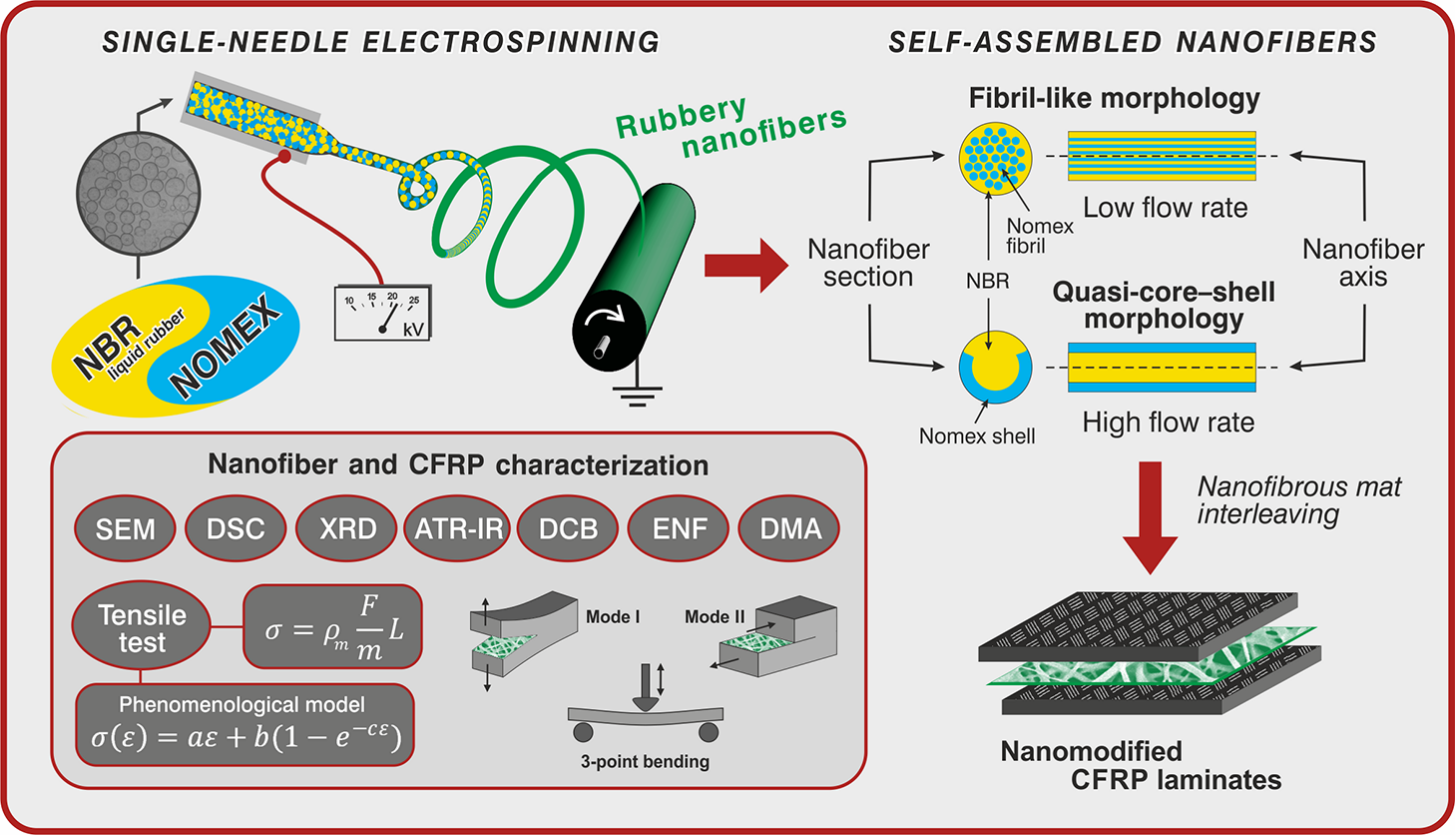
Sketch of the paper rationale: NBR/Nomex self-assembled nanofiber production by the single-needle electrospinning process, mat integration in laminated CFRPs, and panoramic view of performed tests for thoroughly characterizing nanofibrous mats and nanomodified composites.

Figure 1 depicts a sketch of the paper rationale.

## 2 Materials and Methods

### 2.1 Materials

Carboxylated NBR (Nipol 1072CGX) was purchased from Zeon Chemicals [68% mol butadiene (Bu), 28% mol acrylonitrile, and 4% mol methacrylic acid].

Poly(*m*-phenylene isophthalamide) (Nomex) and lithium chloride (LiCl), Sigma-Aldrich, were dried before use in an oven at 110 °C for 3 and 24 h, respectively. *N,N*-Dimethylacetamide (DMAc) and chloroform (CHCl_3_) were purchased from Sigma-Aldrich and were used without any preliminary treatment. The plain weave carbon fiber, 200 g/m^2^, in epoxy matrix prepreg (GG204P IMP503Z-HT, G. Angeloni s.r.l., Venezia, Italy) for composite lamination was kindly supplied by Mind Composites s.r.l., Zola Predosa (Bologna), Italy. The prepreg resin is a bisphenol A diglycidyl ether (DGEBA) epoxy resin, with a resin fraction of 61% on a volume basis, as stated by the technical datasheet.

### 2.2 Solution/Blend Preparation, Nanofibrous Mat Production, and Their Characterization

NBR solution for blend preparation (*s*-NBR_*b*, 10% wt) was prepared in CHCl_3_ (e.g., 1.0 g of polymer in 6.0 mL of solvent) under magnetic stirring at 50 °C heating for at least 4 h until a homogeneous solution is formed. In the solution label, the “*s*” prefix means “solution”, while the “*b*” suffix means that the solution is intended for blend preparation.

Nomex solution for obtaining NBR/Nomex blends (*s*-NX_*b*, 10% wt) was prepared in DMAc in the presence of 3.5% wt of LiCl (e.g., 1.0 g of the polymer and 0.35 g of LiCl in 9.6 mL of solvent). The salt was dissolved in DMAc under magnetic stirring for at least 1.5 h at 80 °C before adding Nomex staples; then, the mixture was stirred at a maximum of 80–90 °C until complete polymer dissolution.

The solution for plain Nomex electrospinning (*s*-NX, 10% wt) was prepared via dilution with CHCl_3_ of a 14% wt Nomex solution and LiCl 3.5% wt in DMAc (*s*-NX_*conc*, e.g., 1.4 g of polymer and 0.35 g of LiCl in 9.2 mL of DMAc, followed by the addition of 2.7 mL of CHCl_3_). The concentrated Nomex solution (*s*-NX_*conc*) was prepared as described above for *s*-NX_*b*. After CHCl_3_ addition, the resulting mixture was stirred for at least 1 h before electrospinning. The *s*-NX solution was not directly prepared in the DMAc/CHCl_3_ solvent system because chloroform is a nonsolvent for the polyaramid; thus, its dissolution does not completely occur, with the polymer swelling mostly.

NBR/Nomex blends were prepared by mixing together *s*-NBR_*b* and *s*-NX_*b* solutions in different proportions (40, 50, and 60% wt of *s*-NBR_*b* solution). Polymer blends were stirred for a minimum of 1.5 h before electrospinning to ensure proper homogenization.

Mats made of mixed nanofibers are labeled *n*-X/Y, where *n* stands for the “nanofibrous mat” and X and Y represent the percentage weight fractions of NBR and Nomex, respectively, in the nanofiber. In the case of mats with 60% wt of NBR, the additional final letter indicates the nanofiber morphology: quasi-core–shell nanofiber (*c*), fibril-like nanofiber (*f*), and a mix of the two morphologies (*m*). Table 1 reports in detail the composition of the solutions and blends.

**Table 1:** Composition Details of NBR and Nomex Solutions and Their Blends

solution/blend	polymer concentration^a^ (% wt)	NBR content (% wt)	Nomex content (% wt)	LiCl (% wt)	solvent system
*s*-NBR_*b*	10.0	10.0			CHCl_3_
*s*-NX_*b*	10.0		10.0	2.5	DMAc
*s*-NX_*conc*	14.0		14.0	3.5	DMAc
*s*-NX	10.0		10.0	2.5	CHCl_3_/DMAc 33:67 wt (24:76 v/v)
*s*-40/60	10.0	4.0	6.0	1.5	CHCl_3_/DMAc 40:60 wt (30:70 v/v)
*s*-50/50	10.0	5.0	5.0	1.3	CHCl_3_/DMAc 50:50 wt (38:62 v/v)
*s*-60/40	10.0	6.0	4.0	1.0	CHCl_3_/DMAc 60:40 wt (49:51 v/v)

a In the case of a blend, the value represents the total polymer concentration.

The *s*-60/40 emulsion morphology was observed after 15, 30, and 90 min of *s*-NBR_*b* and *s*-NX_*b* mixing. The emulsion, stirred for three different times, was deposited on a glass slide to record optical microscopy images.

Nanofibrous mats were produced using a 4-needle electrospinning machine (Spinbow) equipped with 5 mL syringes. Needles (internal diameter, 0.51 mm; length, 55 mm) were joined to syringes via Teflon tubing. Nanofibers were collected on a rotating drum covered with poly(ethylene)-coated paper at a tangential speed of 0.39 m/s (drum diameter: 150 mm; 50 rpm). Mats have final dimensions of approximately 30 × 40 cm and a grammage of 10.3 ± 0.8 g/m^2^, equivalent to thicknesses in the 35–50 μm range. Electrospinning was conducted in the air atmosphere at 23–26 °C and 28–34% relative humidity (RH).

Process parameters are listed in Table 2. The needle-to-collector distance required for the *n*-60/40_*c* mat electrospinning was reduced by ≈40% with respect to the distance required for electrospinning *n*-60/40_*f* and *n*-60/40_*m* membranes uniquely to achieve a stable process.

**Table 2:** Electrospinning Process Parameters and Nanofiber Diameters of Produced Nanofibrous Mats

							nanofiber diameter^b^		
nanofibrous mat	electrospun solution/blend	NBR content in nanofiber (% wt)	flow rate (mL/h)	electric potential (kV)	distance (cm)	electric field^a^ (kV/cm)	as-spun (nm)	after H_2_O washing (nm)	after CHCl_3_ washing (nm)
*n*-NX	*s*-NX	0	0.25	18.0	8.5	2.1	122 ± 37	115 ± 23	111 ± 30
*n*-40/60	*s*-40/60	40	0.80	22.0	8.0	2.8	420 ± 95	418 ± 103	415 ± 103
*n*-50/50	*s*-50/50	50	0.25	18.0	8.5	2.1	438 ± 102	434 ± 111	363 ± 64
*n*-60/40_*f*	*s*-60/40	60	0.20	25.0	17.5	1.4	429 ± 104	438 ± 114	419 ± 97
*n*-60/40_*m*	*s*-60/40	60	0.35	24.0	17.5	1.4	470 ± 131	466 ± 127	424 ± 99
*n*-60/40_*c*	*s*-60/40	60	1.10	25.0	11.0	2.3	447 ± 101	453 ± 121	443 ± 112

a Calculated as the electric potential to distance ratio.

b Average diameter values were calculated from at least 100 diameter measurements.

Nanofibrous mats were analyzed by scanning electron microscopy (SEM) to determine the nanofiber morphology. To investigate the NBR and Nomex disposition in the nanofiber, selective removal of the sole NBR fraction in the mixed nanofibers was carried out via two consecutive washes in CHCl_3_ (1 + 1 h) on a small piece of the mat in a Petri dish, changing the solvent between each wash. All analyzed surfaces were gold-coated in order to make them conductive.

Differential scanning calorimetry (DSC) measurements were carried out on a TA Instruments Q2000 DSC modulated apparatus equipped with an RCS cooling system. Nanofibrous mat samples (10 mg) were first heated to 100 °C for 15 min to remove humidity, then cooled down to −85 °C, and finally heated again at 20 °C/min in a nitrogen atmosphere. NBR in the bulk form was analyzed after just heating from −85 to 200 °C at the same heating rate.

Attenuated total reflection (ATR)-Fourier transform infrared (FT-IR) spectra were recorded using a Bruker Alpha ATR-FTIR spectrometer, acquiring 32 scans from 4000 to 400 cm^–1^, with a resolution of 2 cm^–1^.

Wide-angle X-ray scattering (WAXS) analyses were carried out at room temperature with a PANalytical X’Pert PRO diffractometer equipped with an X’Celerator detector (for ultrafast data collection). A Cu anode was used as an X-ray source (K radiation: λ = 0.15418 nm, 40 kV, 40 mA), and a 1/4° divergence slit was used to collect the data in the 2θ range from 2 to 60°.

Tensile tests of nanofibrous mats were made using a Remet TC-10 testing machine equipped with a 10 N load cell at a 10 mm/min cross-head separation rate. Nanofibrous mat specimens for tensile testing (20 × 45 mm, width and gage length, respectively) were prepared as previously reported.^20,26,27^ Tensile test data were normalized using a reliable method put forward by the authors, based on the specimen mass normalization of the load instead of its cross-sectional area,^27^ according to the following equation:
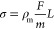
1where σ is the tensile stress, ρ_m_ is the material density (not the apparent membrane density), *F* is the force, *m* is the specimen mass, and *L* is the specimen initial length. In the present case, ρ_m_ has been evaluated as the weighted average value of the two pure polymeric component densities, according to the nanofiber specific composition (see Table S1, Supporting Information S1, for density values). Data were also analyzed using a data fitting model (eq 2), which enables the direct evaluation of the two elastic moduli usually characterizing electrospun nanomats^27^
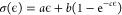
2where *a*, *b*, and *c* are parameters experimentally determined to obtain the data fitting. For each sample type, at least five specimens were tested. The mass-based load normalization method and the data fitting model are extensively discussed in ref (27).

### 2.3 CFRP Production and Characterization

Before integration in CFRPs, nanofibrous mats were washed at room temperature in distilled water to remove LiCl salt. The membranes were immersed twice in water for 10 + 10 min, changing water between each wash and allowing their drying at room temperature. Effective salt removal was assessed via EDX (Energy Dispersive X-ray) analysis.

Specimens for the interlaminar fracture toughness evaluation (DCB and ENF tests) were prepared via hand lay-up, stacking 14 prepreg plies. A single nanofibrous mat in the central interface was embedded besides a Teflon film as a trigger for specimen delamination (Figure S1, Supporting Information S2). Reference panels without the nanofibrous mat were also produced for the sake of comparison.

Specimens for DMA tests were obtained stacking 10 plies of prepregs (Figure S3, Supporting Information S2). All the interfaces (except for the external ones) were nanomodified for a total of nine interleaved mats. Unmodified CFRP specimens were also produced as reference.

Composite panels are labeled C–Z, where C stands for “composite” and Z represents the composition of the abovementioned nanofibrous mat (X/Y, NX). The unmodified composite is labeled C-Ref. Uncured panels underwent a preliminary treatment of 2 h at 45 °C under vacuum for better impregnation of nanofibers prior to the curing cycle in an autoclave (2 h at 135 °C, under vacuum, at 6 bar external pressure, with a heating/cooling ramp of 2 °C/min). Details of the laminate production and CFRP panel/specimen dimensions are reported in Supporting Information S2.

DCB and ENF tests were carried out using a two-column hydraulic universal testing machine (Instron 8033) equipped with a 1 kN load cell. DCB specimens were tested at a 3.0 mm/min cross-head separation rate, while ENF was tested at 1.0 mm/min. At least three specimens for each CFRP sample/delamination mode were tested.

DCB tests were performed for evaluating the energy release rate in Mode I loading (*G*_I_) at both the initial and propagation stages (*G*_I,C_ and *G*_I,R_, respectively) using eq 3(28)
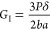
3where *P* is the load, δ is the cross-head displacement, *a* is the crack length, and *b* is the specimen width.

ENF tests were carried out for evaluating the fracture toughness in Mode II loading (*G*_II_) at both the initial and propagation stages (*G*_II,C_ and *G*_II,R_, respectively) using eq 4(29)
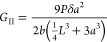
4where *L* is the span length between supports.

The *G*_R_ was evaluated considering a crack length range of 48–80 mm for Mode I and a 32–60 mm range for Mode II tests.

DMA tests were performed using a Netzsch DMA 242 E Artemis in the three-point bending deformation mode (40 mm fixed span support). DMA tests were carried out in the −85–220 °C range at a 3 °C/min heating rate, 1 Hz frequency, 20 μm amplitude, and 1.5 static force/dynamic force ratio.

## 3 Results and Discussion

Currently, single-needle electrospinning of rubbery blends is almost the only way to produce stable NBR rubbery nonwoven mats without additional steps,^20,23^ such as rubber crosslinking,^30^ during or after the electrospinning process. Indeed, pure “liquid” NBR nanofiber production is prevented by the low *T*_g_ of rubber, which leads to film formation. However, as previously demonstrated by the authors,^20^ the use of a semi-crystalline thermoplastic polymer with suitable characteristics, like a low *T*_g_ and a melting temperature (*T*_m_) above room temperature, can be exploited to produce a nanofibrous morphology with elastomeric behavior. The obtained structure resembles the one displayed by TPEs, resulting in dimensionally stable nanofibers, thus overcoming the detrimental rubber cold flow. The blending approach proved to be a smart way to obtain nanofibers with tailored characteristics. Virtually, any type of polymer can be mixed together, provided that they can produce electrospinnable solutions. Even almost immiscible polymers (from a thermodynamic point of view), like the pair NBR/PCL, may be blended and processed via electrospinning without the phase separation phenomenon.^20^ Indeed, the rapid solvent evaporation, faster than solvent casting or spin-coating processes, avoids phase separation. According to the approach proposed by Hoftyzer and van Krevelen,^31^ the miscibility of a polymer pair can be evaluated considering the thermodynamic solubility parameter δ or Hildebrand solubility parameter. This, in turn, derives from three different contributions: the Hansen solubility parameters (δ^2^ = δ_d_^2^ + δ_p_^2^ + δ_h_^2^, where δ_d_ accounts for dispersive forces, δ_p_ relates to polar forces, and δ_h_ accounts for the hydrogen bonding ability). The relative miscibility of the two polymers can thus be evaluated according to the following equation:
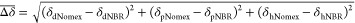
5

When the difference between the δ parameters () for the polymer pair is below 5 MPa^1/2^, there is good miscibility, while 5<  <10 accounts for only partial miscibility.^31,32^

The NBR/Nomex pair has  = 5.7 MPa^1/2^; details about its calculation are reported in Supporting Information S3. This value is slightly higher than the abovementioned threshold, placing the rubber/polyaramid pair into the zone of thermodynamic partial miscibility. However, it is worth mentioning that blending may be still possible, even in this thermodynamically unfavorable situation (see NBR/PCL nanofibers, homogeneously blended with a  = 7.9 MPa^1/2^).^20^ Indeed, kinetic factors may decisively contribute to form a miscible blend since the very fast solvent evaporation occurring during electrospinning can “freeze” a partially miscible pair in a single-phase material.^32^

### 3.1 Morphological and Thermal Characterization of Nanofibrous Mats

The produced nanofibers are characterized by diameters in the 420–470 nm range, except for *n*-NX, which displays a smaller average dimension (122 nm).

The first column of Figure S4, Supporting Information S4, depicts the electrospun nonwovens after washing in water for removing LiCl, required for dissolving the polyaramid (as-spun nanofibers, not shown, display the same morphology as after water washing). The process parameters, as well as complete diameter measurements, are reported in Table 2.

The as-spun and water-washed nanofibers show a smooth surface without defect-like beads, while fiber bundling may occur. Given the critical role played by RH, which at high values favors fiber alignment,^33^ precise humidity control was required during electrospinning. Keeping the RH < 35% allows for a random deposition of nanofibers, as required to obtain a 2D isotropic interlayer reinforcement.

**Figure 2:**
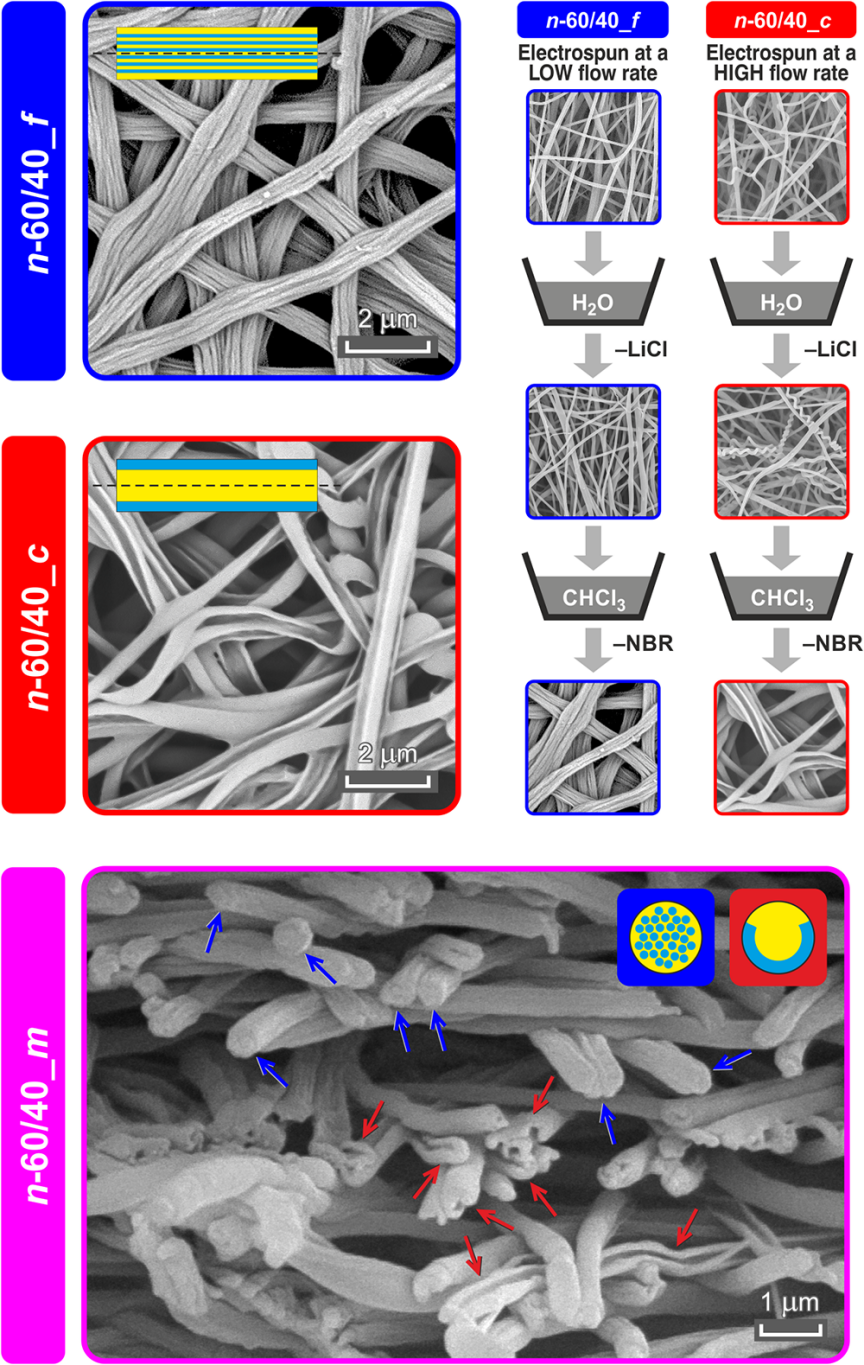
SEM micrographs of *n*-60/40 nanofibrous mats after washing in chloroform to eliminate NBR. For *n*-60/40_*m*, the cross-sectional view is shown (blue arrows: *n*-60/40_*f* nanofiber type, red arrows: *n*-60/40_*c* nanofiber type). On the top right, the washing procedure for LiCl and NBR selective removal is shown.

To investigate the arrangement of the two polymeric components within nanofibers, selective removal of the rubber fraction was carried out by washing mats in chloroform, a nonsolvent for the polyaramid, after washings in distilled water, as shown in Figure 2, top right. A peculiar nanofiber morphology emerged (Figure 2), while insignificant fiber diameter lowering was detected (Table 2).

In particular, the *s*-60/40 solution leads to different morphologies depending on the processing conditions, namely, the applied flow rate (the working distance was also adjusted in the case of *n*-60/40_*c* to attain a stable process). A sketch of the two fiber morphologies is depicted in Figure 1 and also reported along with the SEM images shown in Figure 2. As low as a 0.20 mL/h flow rate gives a “fibril-like” nanofiber (*n*-60/40_*f*). Here, it is possible to distinguish a sort of ultra-thin fiber bundle in the inner section of the tubular structure, which can be safely considered mainly Nomex-based, since they do not solubilize in chloroform, and a layer that surrounds such a core that can be removed upon washing and should thus be mostly NBR. The fibril-like morphology is still present when increasing the flow rate to 0.35 mL/h (*n*-60/40_*m*), but it is combined with a completely different arrangement. The latter morphology resembles a quasi-core–shell structure: Nomex lies in the incomplete outer shell, while NBR lies in the inner channel that upon chloroform washing is removed, leaving a C-shaped polyaramidic residue. The so-called quasi-core–shell morphology becomes the only arrangement when setting a significantly higher flow rate (1.10 mL/h, *n*-60/40_*c*).

All the resulting morphologies imply some degree of self-assembly of NBR and Nomex fractions during electrospinning or even before it, when the solvent is still predominant with respect to the polymeric fraction. Such behavior was already found for immiscible polymers processed via electrospinning. An example is represented by the electrospinning of the polyimide/polyvinylidene fluoride (PI/PVdF) pair, which shows a peculiar “multi-core-shell” morphology.^34^ In the cited work, different diameters of “cores” were obtained by varying the composition of the polymeric fractions. The increase of the PI fraction leads to the formation of biphasic systems where the dispersed phase, made of PI, is comprised of larger drops. As a consequence, the diameter of the multiple cores increases. The formation of continuous PI “filaments”, starting from PI drops, was explained via the simulation of the coalescence phenomena occurring during the electrospinning, mainly due to the flux to which the biphasic system is subjected inside the capillary.^34^

The opaque aspect of NBR/Nomex blends strongly suggests emulsion formation, confirmed by optical micrographs taken at different times of mixing of NBR and Nomex in the CHCl_3_/DMAc solvent system (Figure S5, Supporting Information S5).

The solution morphology changes upon continuous stirring, reaching a more uniform aspect after 90 min. For this reason, the blends were electrospun after stirring for at least 1.5 h after mixing of homopolymer solutions. Under static conditions, the emulsion tends to change the aspect, with features intermediate between 30 and 90 min of stirring morphologies (a deeper investigation of the emulsion stability and its characterization by IR spectroscopy are reported in Supporting Information S5). Moreover, during electrospinning, the emulsion is also subjected to both the pressure and electrostatic field, making the exact interpretation of the phenomenon even more complex. However, it can be concluded that emulsion formation is responsible for the obtained morphologies.^35,36^ Indeed, the electrospinning process parameters have a profound effect on the final morphology, revealing that the combination of a high flow rate (1.10 mL/h) and a high electrostatic field (2.3 kV/cm) favors the formation of a peculiar morphology, highly resembling a core–shell structure (*n*-60/40_*c* membrane).

**Figure 3:**
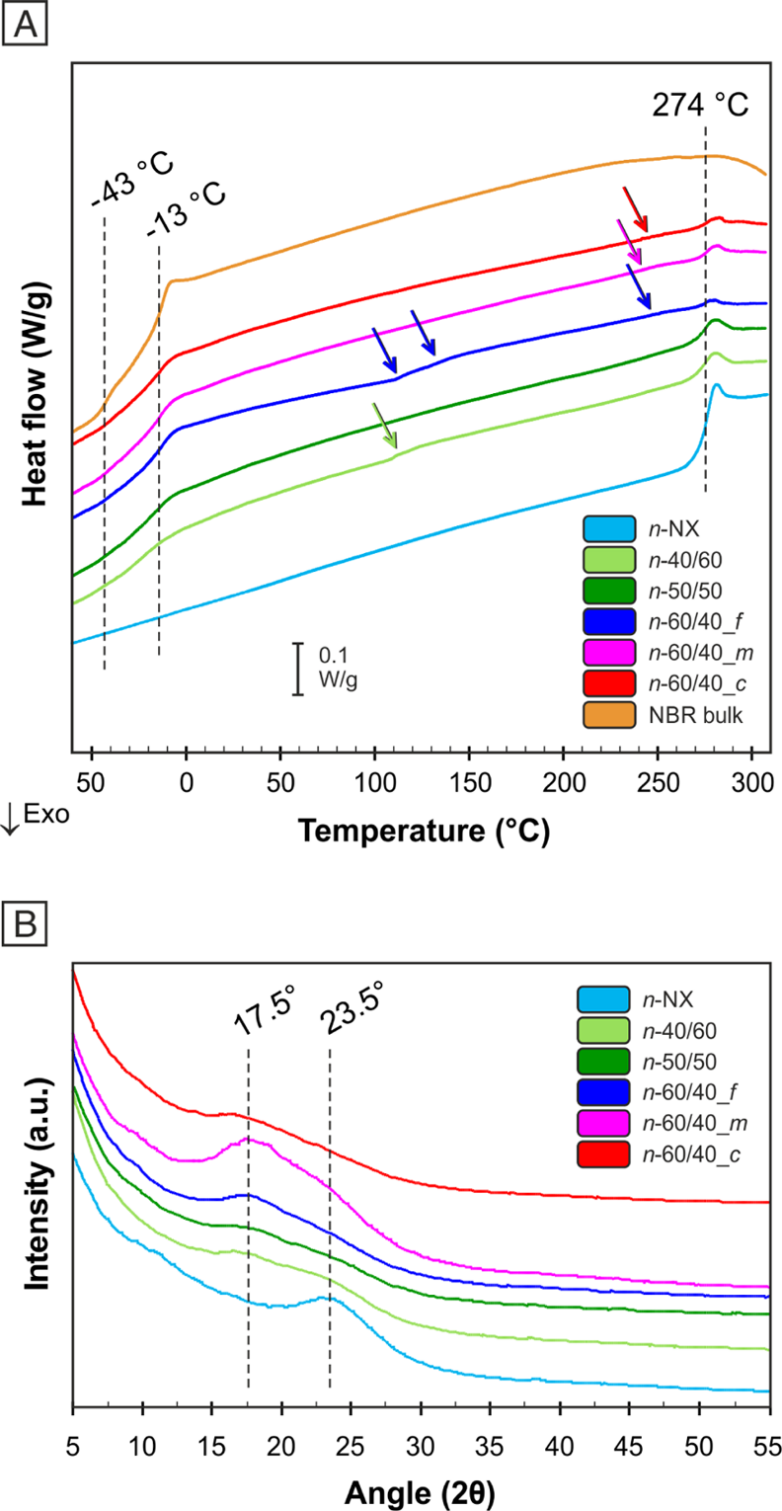
(A) DSC analysis of nanofibrous mats and bulk NBR (the arrows indicate additional glass transitions to those of NBR and Nomex). (B) WAXS diffractograms of nanofibrous mats (after LiCl removal via water washing).

Electrospun mats were thermally characterized via DSC analysis (Figure 3A). The thermograms of NBR/Nomex mats show the presence of both homopolymer *T*_g_s (−43 and −13 °C for bulk NBR, 274 °C for Nomex nanofibers) in all the cases, regardless of the rubber percentage in the nanofiber and the processing conditions. This behavior is completely different with respect to the one previously observed in NBR/PCL nanofibers, where the blends show *T*_g_s in impressive accordance with the Fox equation.^20^ However, all rubbery NBR/Nomex nanofibers show at least one small stepwise signal, ascribable to the glass transition, positioned in between the two *T*_g_ extremes, except for *n*-50/50, where a third glass transition is not detectable. Such evidence suggests that some NBR and Nomex blending can occur, but it does not appear to be the prevailing effect. The evidence that in some cases more than just one additional *T*_g_ is present might be due to the complex evolution of the solution behavior, as also discussed in Supporting Information S5. Indeed, a multiphase situation is observed in the *s*-60/40 initial solution that evolves via the formation of three layers. Two of them are characterized by the concomitant presence of both polymers that could imply the creation of blends with different compositions within the nanofibers. The formation of a miscible blend is maximized in the case of the fibril-like morphology (*n*-60/40_*f*), where the Nomex *T*_g_ is only slightly visible. In this case, polymer mixing is probably favored by an augmented interface area between NBR and Nomex due to the fibril-like morphology.

**Figure 4:**
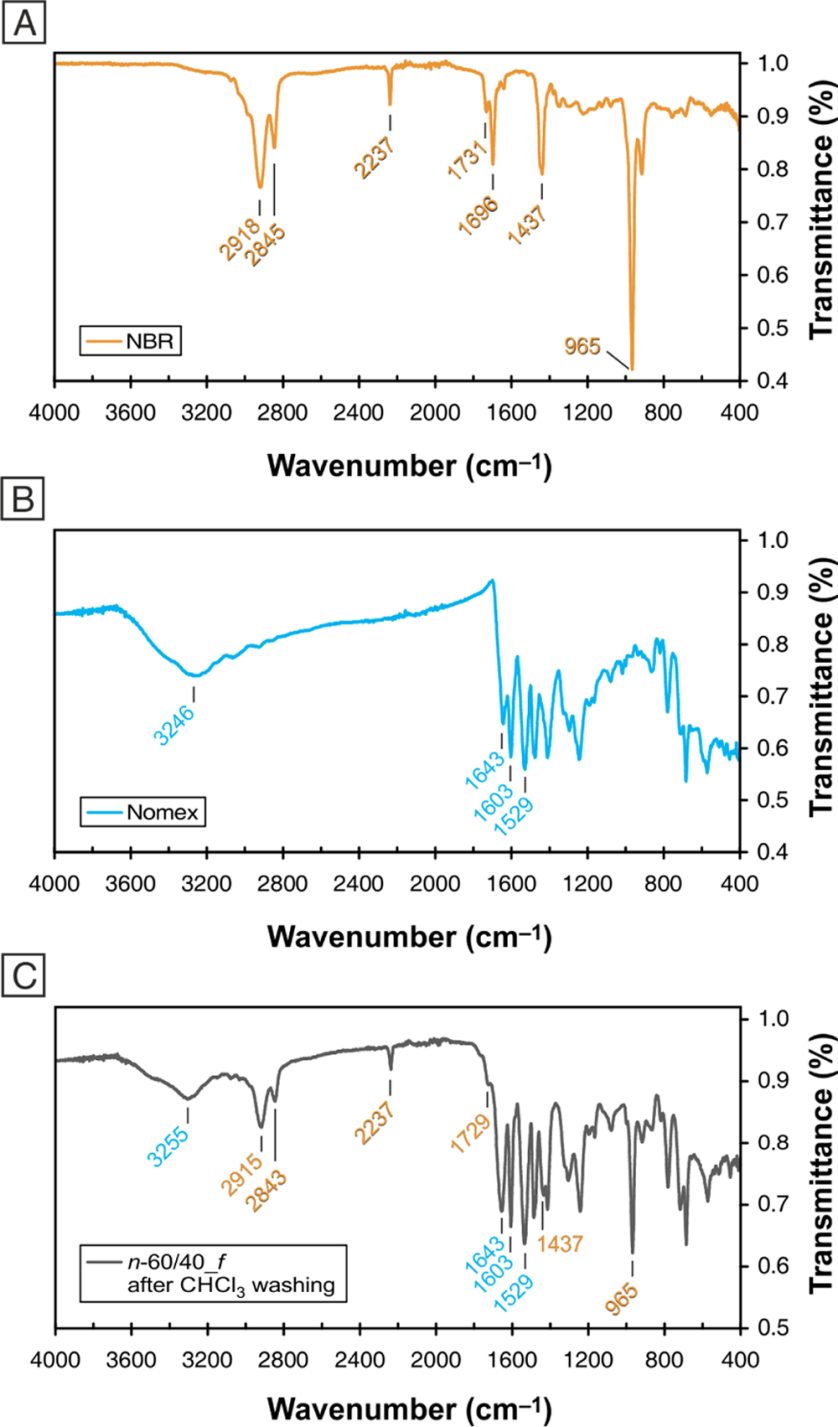
ATR-IR spectra: (A) NBR, (B) Nomex, and (C) *n*-60/40_*f* after CHCl_3_ washing.

The low extent of miscible blend formation is, however, expected for the morphologies elucidated by SEM images, where neat interfaces can be observed (Figure 2). IR spectra of *n*-60/40_*f* and *n*-60/40_*c* mats reveal the presence of NBR even after mat washings in chloroform (see Figure 4C; only the spectrum of *n*-60/40_*f* is displayed, the one of *n*-60/40_*c* is similar). In fact, signals typical of both NBR and Nomex are still present, such as the nitrile stretching at 2237 cm^–1^ for NBR^37,38^ and the amide carbonyl stretching at 1643 cm^–1^ and aromatic C=C stretching at 1603 and 1529 cm^–1^ for Nomex.^39,40^ Such evidence confirms that the nanofiber is composed of an NBR-rich phase that can be removed by chloroform washings, leaving the morphologies reported in Figure 2. However, the resulting fibril-like and quasi-core–shell structures are also made by an NBR-Nomex blend that does not completely release the rubber during washings.

Comparing the  value of the NBR/Nomex pair (5.7 MPa^1/2^) with the one of the NBR/PCL pair (7.9 MPa^1/2^),^20^ from a thermodynamic point of view, the NBR/Nomex pair should produce a more miscible blend than the polymer pair containing the polyester PCL. However, the phase separation occurring in the starting solution (Figure S5, Supporting Information S5) tends to prevent this effect, limiting the interaction of the two polymers: while the electrospinning process plays a key role in forming or not a miscible blend, an actual pre-requisite is a homogeneous mixing of the two polymeric components in the starting solution.

X-ray analysis (Figure 3B) was carried out for investigating the crystallinity of the Nomex fraction inside the nanofiber. Only the Nomex nonwoven shows the reflection at 23.5° typically found in Nomex fibers.^41,42^ Mixed nanofibers, instead, besides displaying Nomex fibrous crystallinity, with a depression of reflection at ≈23.5°, also display a new stronger reflection around 17.5°. In these cases, the development of crystallinity is influenced by both the fast electrospinning process and the presence of NBR. The resulting peak at about 17.5° is indeed found in Nomex films, instead of Nomex fibers, where the strong orientation due to the fiber forming process is lacking and macromolecules may arrange in a different crystal lattice.^43^

### 3.2 Tensile Test of Nanofibrous Mats

The mechanical behavior of rubbery nonwovens, as well as of the Nomex nanofibrous mat, was investigated via tensile testing. Since the mechanism of action of these NBR/Nomex nanofibrous membranes against delamination should be a combination of both matrix toughening and “nanofiber bridging”, the evaluation of the mat mechanical properties is relevant. Indeed, while NBR mixes with the epoxy, acting as a resin toughener, Nomex maintains its original shape even after the curing cycle, providing a three-dimensional network that helps to hamper microcrack formation and propagation.^18^ However, the effectiveness of the so-called “bridging effect” should be correlated to the mechanical properties of the nanostructured interleaf. Consequently, it may lead to a higher reinforcement action for increased mat mechanical performance, provided a good adhesion between the epoxy resin and nanoreinforcement.

**Figure 5:**
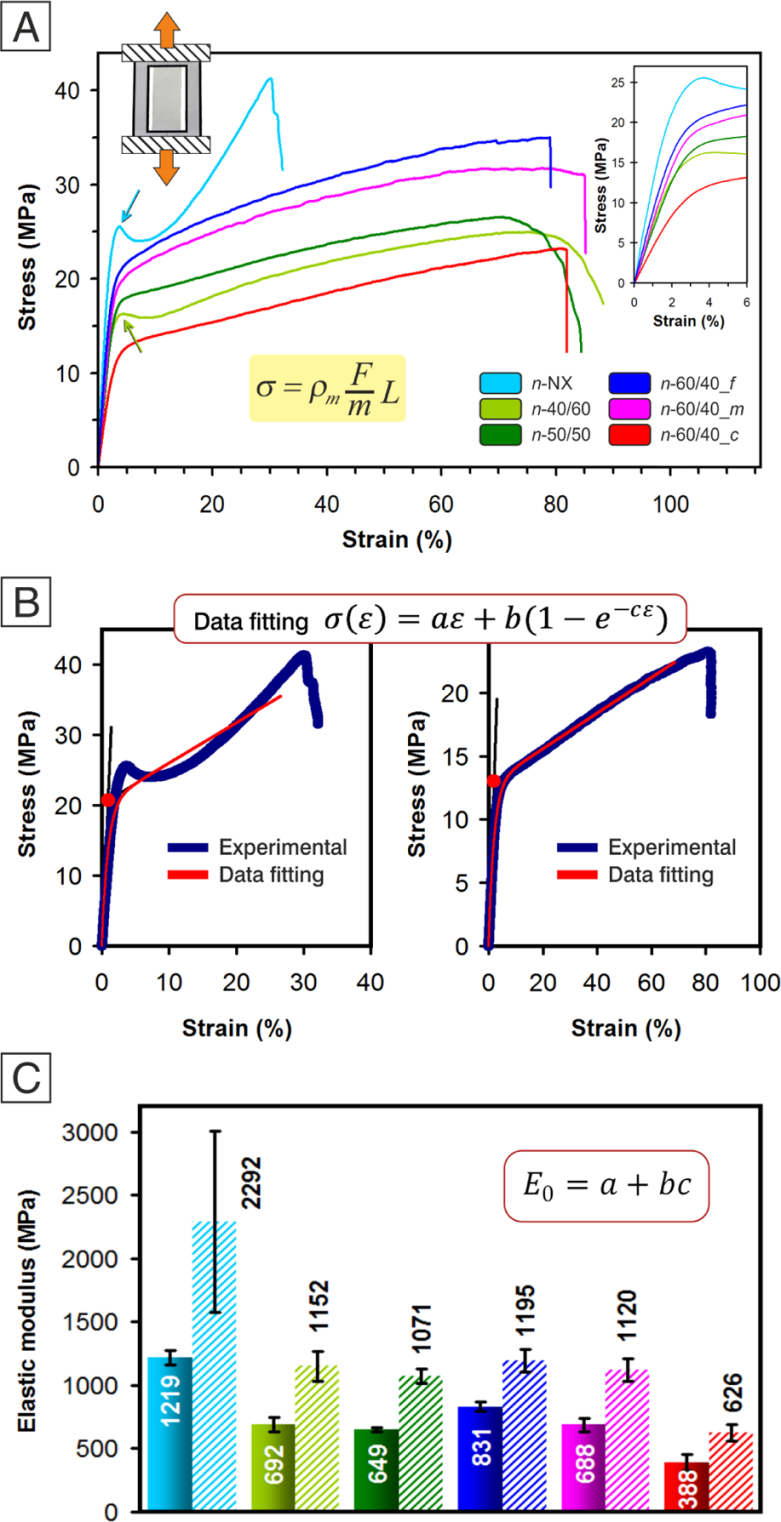
Mechanical characterization of nanofibrous mats. (A) Representative tensile stress–strain curves. The stress is calculated according to eq 1 for obtaining reliable mechanical properties. (B) Examples of application of the data fitting model according to eq 2. (C) Comparison between the elastic modulus calculated from linear regression of stress–strain curves (same values reported in Figure 6C, first bar set) and on the basis of (*a*–*c*) parameters (*E*_0_, second bar set).

Figure 5A reports the tensile stress–strain curves representative of each mat sample.

**Figure 6:**
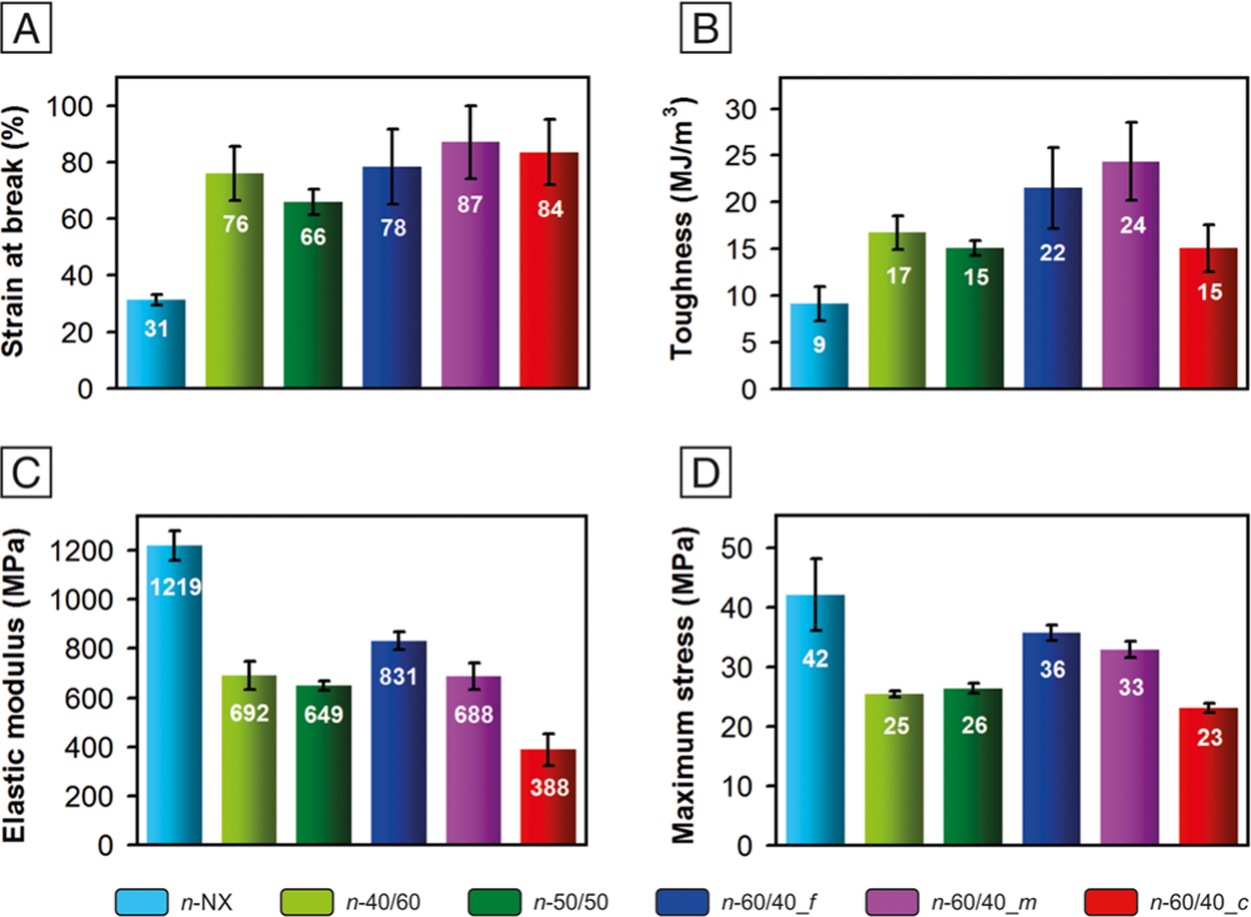
Tensile test results: (A) strain at break, (B) toughness, (C) elastic modulus evaluated as the slope of the linear regression in the 0–1% strain, and (D) maximum stress.

The rubber effect is clearly evident. All NBR/Nomex nonwovens display a ductile behavior, with a maximum strain in the 66–87% range (Figure 6A), while the Nomex mat (*n*-NX) shows a more fragile behavior, characterized by a lower elongation at break (ε_max_ = 31%). As expected, the toughness is significantly higher for rubbery mats, from 1.7 to 2.7 times that of the *n*-NX membrane (Figure 6B).

The presence of the rubber component also affects the mats’ elastic modulus (Figure 6C), resulting in significantly lower than the polyaramid nonwoven one, which is 1219 ± 60 MPa, a value comparable with the one previously observed for randomly oriented Nomex nanofibers.^44^ Such an effect is probably boosted by both partial NBR/Nomex mixing, as confirmed by IR spectra (Figure 4), and by the absence of significant Nomex crystallinity, as revealed by X-ray analysis (Figure 3B). Indeed, if the two polymers completely separate, the actual mechanical performance of the polyaramid would not be so much affected. The mat stiffness does not appear to be sensitive to the actual rubber fraction in the nanofibers, with the elastic modulus in the 650–830 MPa range in all cases but *n*-60/40_*c*, at least in the investigated 40–60% wt NBR range. *n*-60/40_*c* represents an exception: its Young’s modulus (388 ± 64 MPa) is significantly lower than that of the other rubbery mats, suggesting an impact of the nanofibrous morphology on the overall membrane mechanical properties.

Previously, the nanofiber diameter was demonstrated to affect the mats’ mechanical properties through the number of nanofiber crossings, leading to higher mechanical properties and a more fragile behavior at a low nanofiber diameter.^27^ Nomex nanofibers, showing the highest tensile properties, also have the smallest diameter, which is about one-fourth of the rubbery nanofibers. However, the fragile behavior of the polyaramid nonwoven cannot be attributed entirely to a diameter effect, since the rigid macromolecular structure relevantly contributes to the stiffness. By comparing the mechanical behavior of rubber-containing mats, it also appears evident that the “internal” nanofiber morphology may differently affect the final nonwoven mechanical properties, even when the initial proportion of NBR and Nomex is the same. A similar behavior is found by analyzing the maximum stress, which is the highest for Nomex nanofibers (42 ± 6 MPa) and quite similar among the mixed fibers, with *n*-60/40_*c* showing once again the lowest value (23 ± 1 MPa, Figure 6D). It is worth pointing out that the discussed mechanical behaviors are highly reliable, given the application of the mass-based normalization of load, which avoids bias due to mat porosity and mat thickness measurements.^27^ Tensile test data were also analyzed by a data-fitting model (eq 2) previously introduced^27^ and successfully applied to graphene-reinforced nylon 66 nanofibers^26^ and NBR/PCL^20^ nanofibrous mats, showing an impressive fitting ability. While in the cited works the model fits well, regardless of the nanofiber nature (whether it is elastomeric or not), in the present Nomex and NBR/Nomex mixed mats, the model fits well only when at least 50% wt of rubber is present. Indeed, Nomex nanofibers show a stress–strain curve profile that resembles the one shown by bulk dog-bone specimens made of thermoplastics. In particular, the yield point is generally not present when dealing with nanofibrous mats,^20,23,26,27,45−50^ except for a few reported cases.^51,52^

The presence of a yield point or a sharp change of the curve slope moving from stage I (initial nonlinear trend) to the stage II (linear trend) zone does not allow a correct application of the fitting model. For a detailed description of the tensile curve behavior and the stages in which the material behavior can be divided, see ref (27). By increasing the rubber content in the nanofiber, the yield point becomes less evident, and the data fitting improves. Therefore, it can be concluded that when the transition between stages I and II is “soft”, the fitting model can be applied correctly and used for evaluating the two elastic moduli, which generally characterize the stress–strain curves of nanofibrous mats.^20,26,27^

In Figure 5B, two representative cases, that is, the worst and the best exempla of data fitting model application, are reported, while complete data fitting results can be found in Supporting Information S6.

Histograms of Figure 5C show the comparison between the elastic moduli (*E*) calculated as the slope of the tangent to the stress–strain curve in the 0–1% deformation range and on the basis of the parameters *a*, *b*, and *c* used for the data fitting. In the latter case, the calculated *E*_0_ values are significantly higher than *E* ones, still displaying a similar trend. Since *E*_0_ represents the extrapolation of the tensile modulus for ε → 0, this behavior is expected, as already found for randomly oriented nylon 66 electrospun membranes.^27^ In the case of the *n*-NX mat, a high standard deviation can be noted (a coefficient of variation of 31% vs < 11 of the other NBR/Nomex mats) as a consequence of the cited poor data fitting.

### 3.3 Mode I and Mode II Interlaminar Fracture Toughness Evaluation of Nanomodified CFRPs

The interlaminar fracture resistance of CFRPs was assessed via DCB and ENF tests, where the laminate is solicited in Mode I and Mode II loading modes, respectively. Figure S2 in Supporting Information S2 shows a schematic representation of DCB and ENF specimens.

In the first case, the specimen beams are subjected to a perpendicular load with respect to the crack propagation plane, while in the second one, a bending deformation is imposed to simulate the sliding of the two constituent beams. The energy release rate (*G*), calculated from the delamination tests, can be ascribable to two different crack propagation stages: the initiation stage (*G*_C_), in which the delamination onset starts from the Teflon-initiated artificial crack, and the propagation stage (*G*_R_). Mode I and Mode II results are summarized in Table 3.

**Table 3:** Mode I (DCB Test) and Mode II (ENF Test) Results: Maximum Load and Energy Release Rate Calculated at Initial (*G*_I,C_ and *G*_II,C_) and Propagation (*G*_I,R_ and *G*_II,R_) Stages

CFRP	Mode I loading test (DCB)			Mode II loading test (ENF)		
	max load (N)	*G*_I,C_ (J/m^2^)	*G*_I,R_ (J/m^2^)	max load (N)	*G*_II,C_ (J/m^2^)	*G*_II,R_ (J/m^2^)
C-Ref	46 ± 5	517 ± 106	558 ± 65	659 ± 54	2261 ± 131	2748 ± 231
C-NX	33 ± 2	255 ± 7	188 ± 2	723 ± 27	2089 ± 162	2637 ± 261
C-40/60	57 ± 2	792 ± 213	887 ± 70	747 ± 44	2546 ± 513	3222 ± 352
C-50/50	61 ± 1	804 ± 94	1028 ± 76	756 ± 31	2750 ± 366	3223 ± 413
C-60/40_*f*	64 ± 5	910 ± 55	1161 ± 155	791 ± 22	3466 ± 354	3830 ± 399
C-60/40_*m*	64 ± 1	880 ± 136	1252 ± 162	760 ± 9	2864 ± 213	3466 ± 354
C-60/40_*c*	73 ± 3	1102 ± 107	1559 ± 238	752 ± 14	2812 ± 412	3302 ± 439

**Figure 7:**
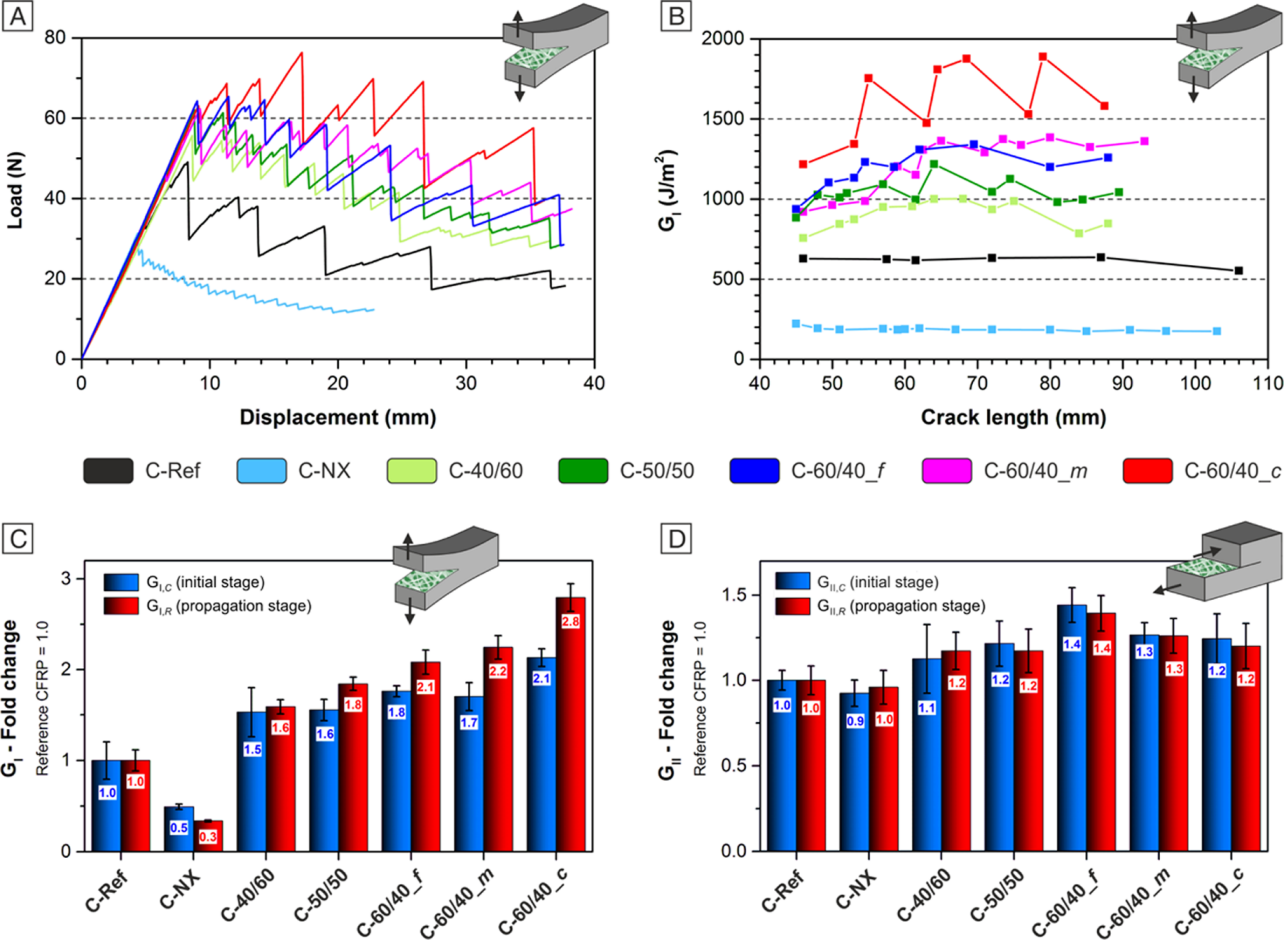
DCB test results: (A) load–displacement curves, (B) *R*-curves related to the same specimens displayed in (A), and (C) average *G*_I_ fold change. ENF test results: average *G*_II_ fold change (D). Bars in (C,D) are expressed as the relative value variation with respect to the reference sample (C-Ref), whose value is set as 1.0.

Figure 7A displays representative load versus displacement curves derived from DCB tests. These trends give a first indication of the CFRP delamination behavior.

Laminates with interleaved mixed fiber nanomats show the same trend and the slope of the corresponding reference CFRP until the first force drop occurs. Nevertheless, the crack initiation is postponed, and the maximum force is increased (up to +59% for C-60/40_*c* sample). On the contrary, 100% Nomex nanofibers clearly promote the delamination phenomenon (C-NX sample). Indeed, the maximum force is significantly lower than the reference one (−27%) besides a quasi-continuous crack propagation, as highlighted by the load–displacement profile characterized by frequent and low-entity load drops. The presence of NBR seems to be able to not only counterbalance the lousy performance of Nomex nanofibers but also impart an overall positive action toward delamination.

Assuming that the NBR-only fraction, mixing with the epoxy resin, gives the most contribution against delamination, some positive effect may arise from the rubber fraction mixed with the polyaramid (as assessed by IR, Figure 4C), favoring its compatibility with the hosting epoxy resin.

*R*-curves (fracture toughness vs crack length) calculated from the Mode I loading tests are shown in Figure 7B. As for the maximum load, *G*_I_ trends show a significant ability of NBR/Nomex nanofibrous mats to hinder delamination: the energy required for the crack propagation is considerably higher than the unmodified reference (up to +180%, Figure 7C). A 40% wt rubber content in the nanofiber increases the *G*_I_ by 50–60% at both initiation and propagation stages (C-40/60). Increasing the NBR to 50% wt (C-50/50) leads to further *G*_I__,__R_ enhancement (+84% with respect to C-Ref), while *G*_I__,__C_ (+55%) is similar to the C-40/60 one. Moving to nanofibers with a prevalent rubber fraction (60% wt of NBR), the laminate toughening is even better, also showing a *G*_I_ dependence from the nanofiber morphology. In particular, the mat composed of quasi-core–shell nanofibers (C-60/40_*c*) enables the best overall CFRP performance: +113% of *G*_I__,__C_ and +180% of *G*_I__,__R_ compared to unreinforced C-Ref. Instead, the other two morphologies (fibril-like nanofibers and a mixture of fibril-like and quasi-core–shell nanofibers) have quite a similar impact toward delamination resistance, whose modified CFRPs (C-60/40_*f* and C-60/40_*m*) show an increase of both *G*_I,C_ and *G*_I,R_ in the 70–75 and 108–124% range, respectively.

The two fibrous morphologies with the same 60% wt of rubber content can account for the different reinforcing actions. Quasi-core–shell nanofibers can be considered a rubber reservoir that can toughen the epoxy resin more effectively than the fibril-like nanofiber morphology. SEM micrographs of the delamination surfaces after DCB tests confirm this hypothesis, as discussed later at the end of this section.

Comparing these results with previously reported nanomodified CFRPs with NBR/PCL nanofibrous mats,^21^ it is evident that the enhancement is less pronounced (up to +180 vs +480%) but still highly significant. However, the addition of NBR/Nomex mixed nanofibers does not cause any important *T*_g_ lowering or stiffness reduction of the laminate, as demonstrated by DMA discussed in Section 3.4.

The Nomex nanofibrous mat worsens the overall delamination performance, lowering the interlaminar fracture toughness. A significant reduction of both *G*_I,C_ (−51%) and *G*_I,R_ (−67%) is observed, precluding the use of meta-aramidic nanofibers on themselves as epoxy composite tougheners, since they act very similar to a bulk film with poor adhesion to the matrix. The bad performance of Nomex nanofibers should derive from poor adhesion with the epoxy resin. Since (aliphatic) polyamide nanofibers are among the most used for reinforcing the interlaminar region of composite laminates,^18^ the detrimental action may be due to the presence of aromatic rings. Moreover, a previous study^53^ demonstrated that the epoxy resin crosslinking is affected by the presence of Nomex nanofibers, which delay or partially hamper the curing process. The latter assumption is confirmed by DMA tests. Concluding, the two detrimental effects may both contribute to the reduced performance of the Nomex-modified laminate.

Regarding ENF tests, the *G*_II_ enhancement is less pronounced but still significant (+40% in the best case, C-60/40_*f*). Analyzing the average *G*_II_ fold change values (Figure 7D), there is, once again, a positive trend by increasing the rubber content. The nanofibrous morphology also impacts the Mode II delamination behavior, affecting the interlaminar fracture toughness in a way opposite to what is observed for DCB tests: the fibril-like morphology provides the best reinforcement. Load-displacement diagrams and *R*-curves of ENF tests are reported in Figure S9, Supporting Information S7.

**Figure 8:**
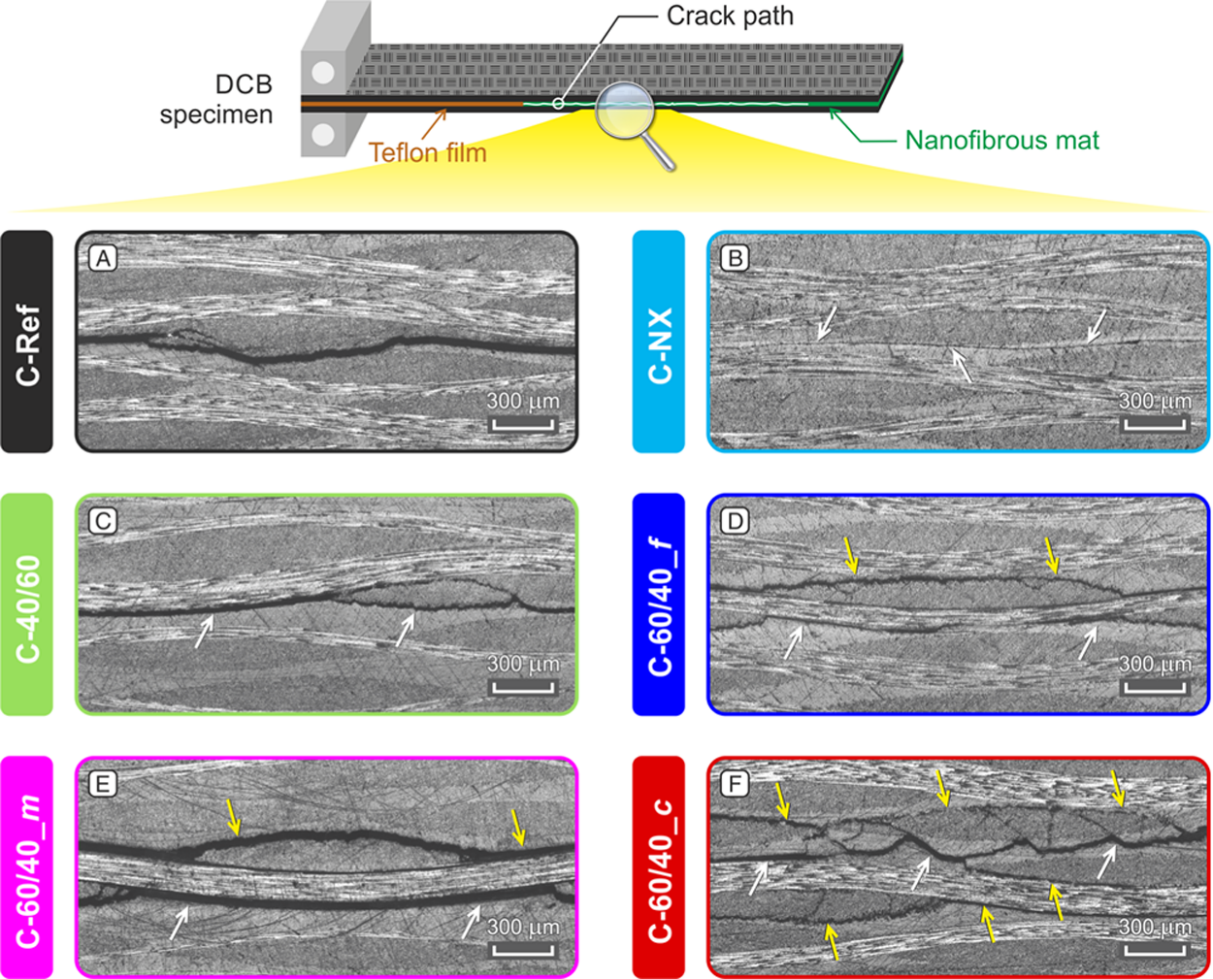
Micrographs of DCB specimens after the delamination tests of nanomodified CFRPs (B–F) and reference laminate (A). White arrows: designed crack plane (central plane) and yellow arrows: plane(s) adjacent to the central one.

Micrographs of tested DCB specimens evidence the strong toughening action of rubbery nanofiber-modified CFRPs: the crack paths are uneven, and they interest more planes than the central one where nanofibers are placed (Figure 8D–F). This behavior is independent of the actual nanofibrous morphology or the rubber content in the nanofiber if NBR is ≥ 50% wt (the C-50/50 micrograph is reported in Figure S10, Supporting Information S7). On the contrary, where Nomex-only nanofibers are used, the crack path is hard to detect due to the “linear” and regular crack propagation, confirming the detrimental performance given by the pure meta-aramid nanomat (Figure 8B). Regarding the modification with nanofibers having less than 50% wt of NBR (C-40/60), the laminate exhibits a more complex crack path than C-Ref. However, the formation of adjacent crack planes to the central one is not observed (Figure 8C).

**Figure 9:**
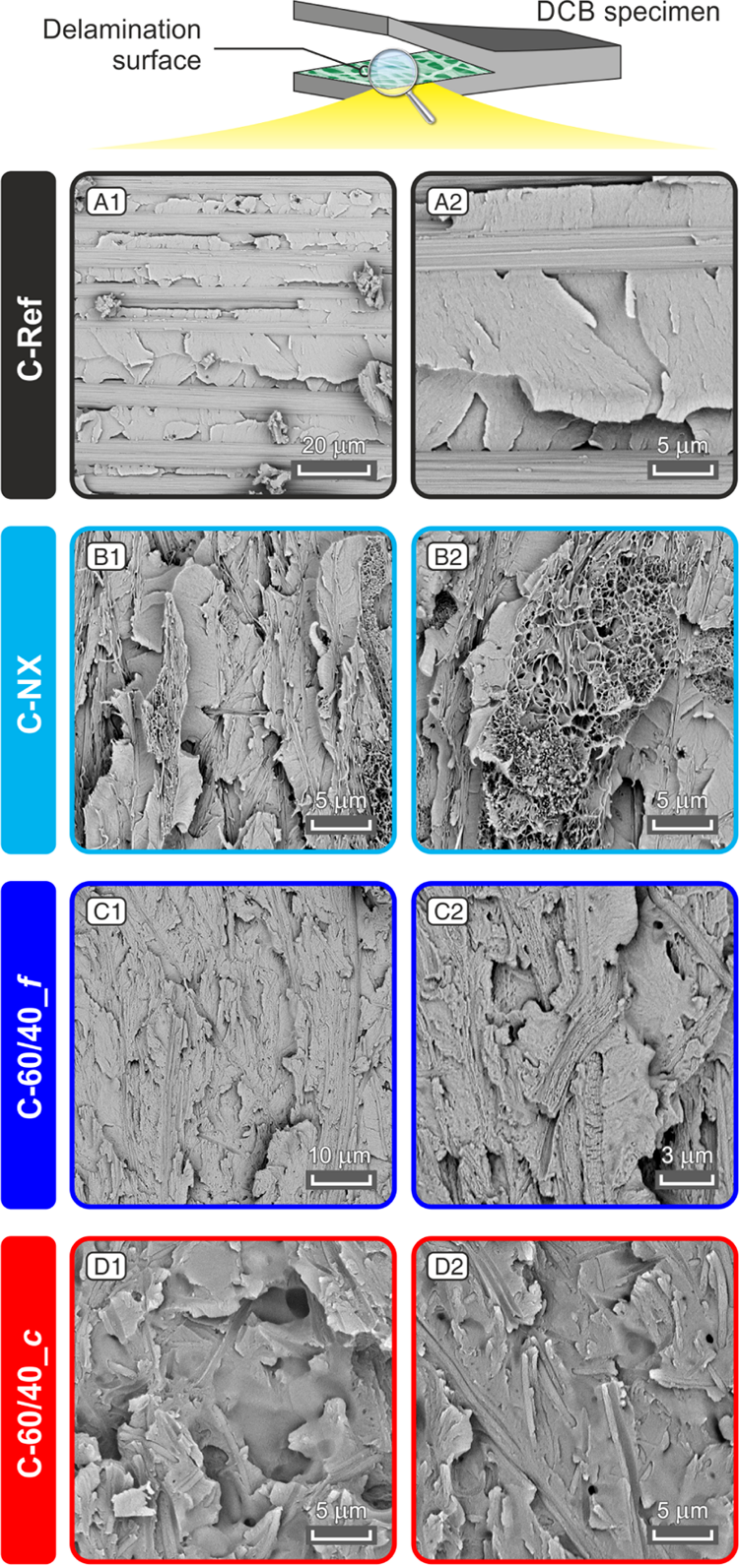
Micrographs of DCB specimens after the delamination tests (A1–D1 and A2–D2): morphology of delamination surfaces showing details of the matrix morphology and nanofibrous mat (where present).

The different delamination behavior is also confirmed by delamination crack surface analysis (Figures 9 and S11, Supporting Information S7, where additional images are displayed).

The unmodified laminate (C-Ref) displays the matrix arranged in wide flat planes, typical of brittle fracture of epoxies. On the other hand, the Nomex-modified CFRP (C-NX) has an overall morphology similar to that of C-Ref, with flat planes originating from fragile matrix fracture. As expected from the Nomex thermal properties (*T*_g_ = 274 °C), the nanofibers maintain the morphology after the curing cycle (at 135 °C), resulting in a clearly visible nanostructure. In some regions, the 3D nanofibrous network appears almost not impregnated (Figure 9B1,B2), supporting the hypothesis that low meta-aramid adhesion with the epoxy matrix is responsible for the bad interlaminar mechanical performance. Moreover, analyzing the C-NX delamination surfaces, regions where the crack does not cross the nanofiber can be found (Figure S11B1, Supporting Information S7).

Analyzing the fracture surfaces of CFRPs modified with 60% wt NBR nanofibers (Figure 9C,D), the situation appears different: in place of completely brittle, smooth, and sharp flat planes, whose morphology is still evident, the surface of epoxy is rougher, indicating that matrix toughening occurred. In particular, C-60/40_*c* shows localized but disseminated regions where the epoxy toughening is more pronounced than others (Figure 9D1). This is probably due to the NBR contained in the nanofiber “core”, released from the Nomex C-shaped reservoir upon curing, allowing improved matrix toughening of localized epoxy volumes. Indeed, this phenomenon is absent in C-60/40_*f* fracture surfaces, where fibril-like nanofibers were interleaved (Figure 9C), as well as in C-40/60 (Figure S11C, Supporting Information S7) and C-50/50 (not shown, but similar to C-40/60). C-60/40_*m* (not shown) displays characteristics of both C-60/40_*f* and C-60/40_*c* delamination surfaces. As revealed by DCB tests, quasi-core–shell nanofibers guarantee the best delamination hindering in Mode I, suggesting that the NBR disponibility to mix with the hosting matrix has an important role. An opposite situation happens when soliciting laminates in Mode II: in this case, the presence of regions with extensive rubber toughening should be avoided.

### 3.4 Thermomechanical Properties of Nanomodified CFRPs

**Figure 10:**
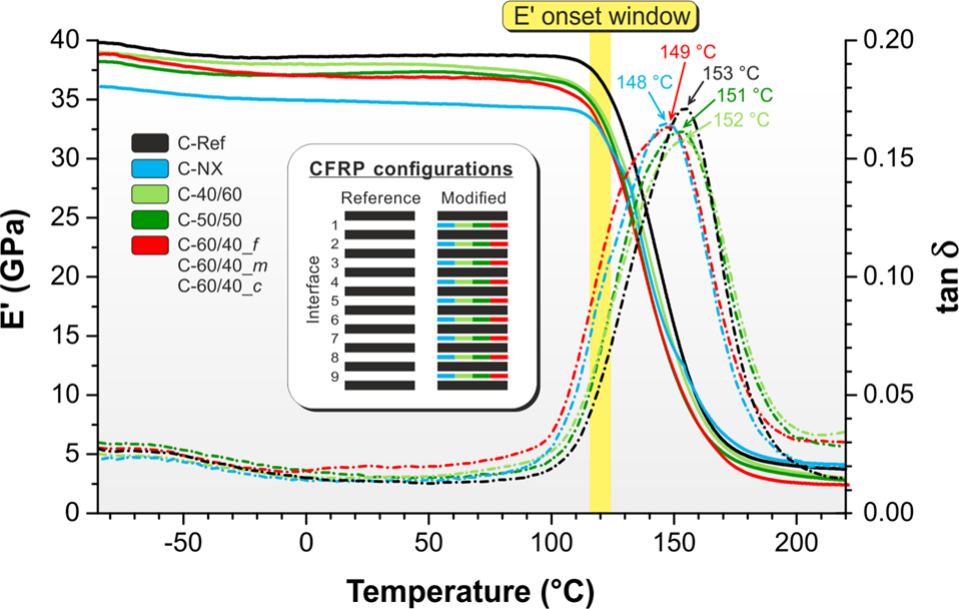
*E*′ (solid lines) and tanδ (dashed lines) representative curves of CFRP samples from −85 to 220 °C, tested at a 1 Hz frequency. The inset graphics shows the CFRP configurations tested by DMA. Only one curve for C-60/40_*c*, C-60/40_*m*, and C-60/40_*f* is displayed for better clarity (all CFRPs modified with mats containing 60% wt of NBR behave in the same way).

The evaluation of the laminate thermomechanical properties is of paramount importance to thoroughly define the overall material’s behavior and, consequently, its application field. In fact, stiffness and/or *T*_g_ lowering represent common drawbacks that can afflict laminates modified with low mechanical and thermal properties materials, like rubbery nanofibers. In our previous work, in the face of a relevant delamination resistance improvement (up to +480% in *G*_I_), NBR/PCL blend nanofibers can significantly affect the laminate thermomechanical properties, particularly the *T*_g_. Therefore, a careful evaluation of the nanomodification extent was suggested to meet the best compromise between mechanical reinforcement and thermomechanical properties.^21^ In the present case, DMA reveals a very slight mat impact on the nanomodified composites (Figure 10 and Table S4 in Supporting Information S7).

The *E*′ onset window is in the range of 115–122 °C, a very tight temperature interval for the extensive nanomodification done on the analyzed laminates. Surprisingly, the worst performance is given by Nomex nanofibers, which are not expected to impact matrix properties but whose lousy interaction with the epoxy resin detrimentally affects the mechanical performance in different points of view. On the contrary, all mixed nanofibers are in the 118–121 °C range, values very close to the reference laminate (122 °C).

Nomex nanofibers negatively affect the composite thermomechanical properties, causing the *E*′ lowering at 25 °C from 39 GPa of unmodified CFRP to 35 GPa, without improving the other tested mechanical properties. It happens even if the polyaramid is well below its *T*_g_; in this situation, no influence on CFRP stiffness should take place, as seen with the interleaved nylon 66 nanofibrous mat (*T*_g_ ≈ 65 °C^26^).^21^ Therefore, the behavior found is probably ascribable to the polyaramid influence on the epoxy crosslinking kinetics, which is somehow hampered.^53^ Such a hypothesis is also confirmed by the slight shift toward a lower temperature of the CFRP tanδ peak (148 vs 153 °C of C-Ref).

All rubbery nanofiber-modified CFRPs, even with 60% wt of NBR, maintain almost all the composite original stiffness (*E*′ below *T*_g_ of 37–38 GPa vs 39 GPa of C-Ref), while the *T*_g_ evaluated at *E*′ onset is only slightly lowered (−5 °C in the worst case of C-60/40_*c*). Therefore, the material maximum operating temperature stays practically unchanged with respect to C-ref, allowing the use of modified laminates in the same application fields of the unmodified commercial CFRP. Laminates reinforced by NBR/PCL nanofibrous mats ended up being highly sensitive to the rubber percentage in the nanofiber, causing an important lowering of the material *T*_g_. In NBR/Nomex mixed nanofibers, the presence of the thermal resistant Nomex instead of the low-*T*_g_ (≈−60 °C) and low-*T*_m_ (≈60 °C) PCL counterpart appears to be fundamental to the retention of CFRP thermomechanical properties.

The analysis of tanδ is useful for damping evaluation since it accounts for the material ability to hamper vibrations via energy dissipation. Tanδ values reveal about +50% in the 20–100 °C temperature range for C-60/40 laminates, regardless of the particular nanofiber morphology. Instead, a small NBR content leads to a smaller effect. All the tanδ curves of rubbery nanofiber-modified CFRPs display a single relaxation associated with the glass transition, with a tanδ peak (*T*_α_ in the 149–152 °C range) very close to the C-Ref one (153 °C). The relaxations display broader peaks due to the plasticizing effect caused by the nanomodification: mixed nanofibers add some plasticization, thanks to the NBR fraction mixing with the resin, while in the case of pure Nomex nanofibers, the plasticizing effect is probably due to the already mentioned influence on the epoxy crosslinking. Indeed, the tanδ curve slightly shifts toward lower temperatures. Moreover, analyzing the full width at half-maximum (FWHM) of the tanδ peaks, one of the Nomex-modified CFRPs is also higher than the one displayed by the reference laminate. Since Nomex cannot mix with the matrix (Nomex *T*_g_ = 274 °C), the found CFRP behavior should be related to the less ability of the epoxy resin to covalent bonding. This causes the formation of local regions (near the interleaved mat) with lower thermomechanical properties.

DMA demonstrates that the integration of NBR/Nomex nanofibrous mats does not reduce the laminate stiffness or its *T*_g_, which stay practically unchanged with respect to the unmodified CFRP. All the original laminate thermomechanical properties are maintained, while benefiting an excellent improvement of the interlaminar fracture toughness.

## 4 Conclusions

NBR/Nomex rubbery nanofibers provide an outstanding reinforcement of the composite interlaminar region, raising the intrinsic safety of nanomodified CFRPs to a higher level. Indeed, Mode I and Mode II loading tests significantly improve the interlaminar fracture toughness, especially *G*_I_ (up to +180%). By contrast, pure Nomex nanofibers dramatically worsen the delamination resistance, suggesting a poor adhesion with the matrix.

NBR/Nomex mixed nanofibers with 40–60% wt of rubber were produced via single-needle electrospinning, without the need for rubber crosslinking to maintain the nanostructure. NBR and Nomex disposition in the nanofiber was investigated, revealing the formation of particular self-assembled structures. By simply acting on electrospinning process parameters, it is possible to obtain two very different morphologies. The application of a low flow rate (0.20 mL/h) leads to the formation of fibril-like nanofibers, where a sort of ultra-thin Nomex fiber bundle stays in the core, surrounded by an NBR layer. Upon increasing the flow rate to 1.10 mL/h, the attained morphology is completely different: it resembles a quasi-core–shell structure, with Nomex in the incomplete outer shell and NBR in the inner channel. The two nanofiber morphologies improve the delamination resistance differently, also suggesting that the way the rubber is located in the nanofibers plays a role in the toughening action. Moreover, NBR/Nomex nanofibers do not lower the laminate thermomechanical properties, as often happens when soft materials, like rubbery ones, are integrated. Indeed, both the original laminate stiffness and glass-transition temperature (*T*_g_) are maintained.

Such evidence paves the way to extensive and reliable use of NBR/Nomex lightweight rubbery mats in composite laminates for improving the delamination resistance without affecting other relevant properties.

## Supporting Information Available

Production and characterization of CFRP laminates; evaluation of the solubility parameters for NBR and Nomex and their miscibility; nanofibrous mat morphologies before and after chloroform washings; IR investigation on the NBR/Nomex emulsion; stress–strain data fitting model; and DCB, ENF, and DMA tests (PDF)
