# Supplementary material for: Self-Assembled NBR/Nomex Nanofibers as Lightweight Rubbery Nonwovens for Hindering Delamination in Epoxy CFRPs
Source: ACS Appl Mater Interfaces. 2021 Dec 23;14(1):1885–99. doi: 10.1021/acsami.1c17643 (PMC8763375; doi:10.1021/acsami.1c17643)
Supplement: Supplementary file 1 — am1c17643_si_001.pdf [file am1c17643_si_001.pdf]

Supporting Information

Self-Assembled NBR/Nomex Nanofibers as Lightweight Rubbery Nonwovens for Hindering Delamination in Epoxy CFRPs

Emanuele Maccaferri[a,], Laura Mazzocchetti[a,b], Tiziana Benelli[a,b], Tommaso Maria Brugo[b,c], Andrea Zucchelli[b,c], Loris Giorgini[a,b]*

[a] Department of Industrial Chemistry "Toso Montanari", University of Bologna, Viale Risorgimento 4, 40136 Bologna, Italy.

[b] Interdepartmental Center for Industrial Research on Advanced Applications in Mechanical Engineering and Materials Technology, CIRI-MAM, University of Bologna, Viale Risorgimento 2, 40136 Bologna, Italy.

[c] Department of Industrial Engineering, University of Bologna, Viale Risorgimento 2, 40136 Bologna, Italy.

Corresponding author's e-mail: emanuele.maccaferri3@unibo.it

S1 – Evaluation of the NBR/Nomex blends density

The density values (ρ_m) of the NBR/Nomex nanofibers required for the correct application of the Equation 1 for the stress (σ) calculation were obtained by applying the so-called rule of mixtures.

The NBR density is 0.98 g/cm^3, the Nomex one is 1.38 g/cm^3. In Table S1 are reported the calculated density of the blends.

Table S1. Density evaluation of polymer blends.

**NBR fraction** (g/g)	**Nomex fraction** (g/g)	**Blend density** (g/cm^3)
0.40	0.60	1.22
0.50	0.50	1.18
0.60	0.40	1.14

S2 - Production and characterization of CFRP laminates

The hand lay-up was carried out in an air-conditioned room (20-22 °C, 23-25% of relative humidity). The nanofibrous mats were directly applied with their paper substrate onto the prepreg during the lamination process. The supporting paper was removed before adding additional prepreg plies. Uncured panels underwent a preliminary treatment of 2 h at 45 °C under vacuum for better impregnation of nanofibers prior curing cycle in autoclave (2 h at 135 °C, under vacuum, 6 bar external pressure, heating/cooling ramp 2 °C/min).

Regarding DCB and ENF tests, CFRP panels (140 × 190 mm) are constituted by 14 plies in total. The specimens were obtained by cutting out the CFRP panel, discarding the edge parts (minimum 15 mm) for avoiding any inhomogeneity.

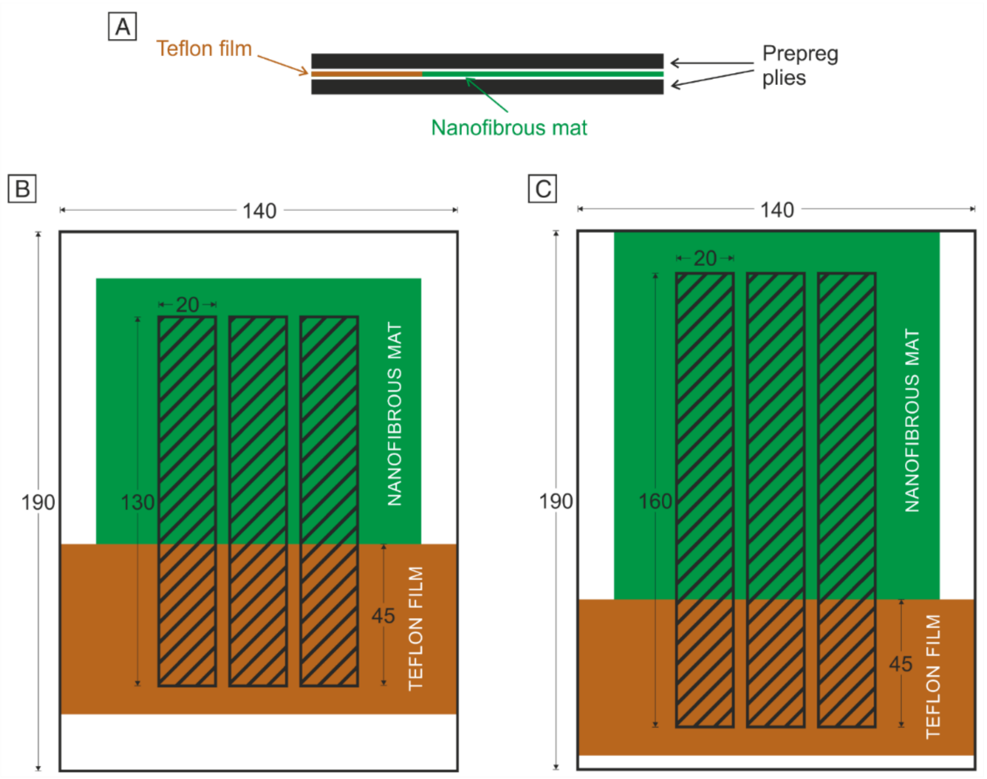

Figure S1. Samples for evaluating the delamination resistance: section view (A) and dimensions (in mm) of CFRP panels and specimens for DCB (B) and ENF (C) tests.

DCB specimens had the following final dimensions: 130 mm total length (L), 20 mm width (b), 45 mm crack length (a), and were tested under a 3.0 mm/min crosshead separation rate. Aluminium blocks were fixed with epoxy resin glue on the tip for the application of the load.

ENF specimens had the following dimensions: 160 mm total length, 20 mm width (b), 45 mm crack length, and were tested under a 1.0 mm/min crosshead separation rate. ENF tests were carried out with 100 mm span ($2L$) between supports, and the specimen placed in the 3-point bending geometry as follows: 50 mm specimen half-span (L) and 30 mm delamination length (a_0).

The G_R was evaluated considering a crack length range of 48-80 mm for Mode I and 32-60 mm range for Mode II tests.

Figure S2 shows a schematic representation of DCB and ENF specimens.

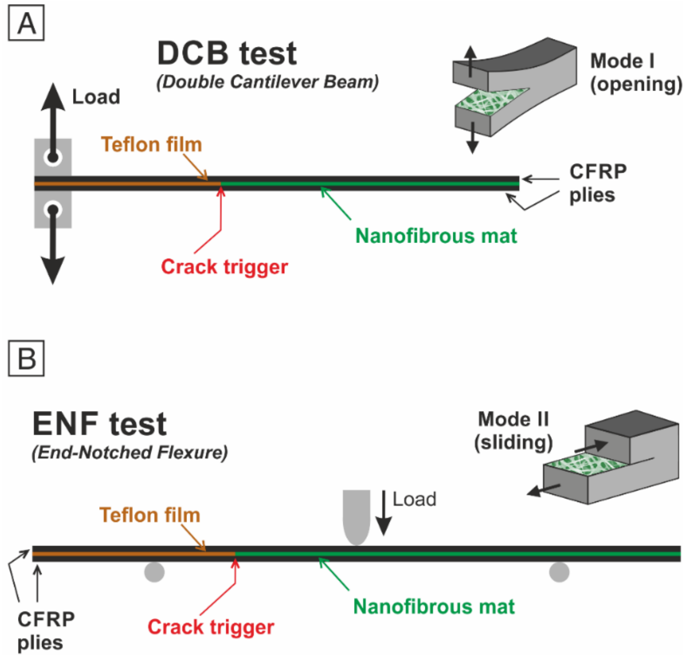

Figure S2. Schematic representation of specimens for the interlaminar fracture toughness evaluation in Mode I (A) and Mode II (B).

Specimens for Dynamic Mechanical Analysis (DMA) were 50 × 8 mm, obtained by cutting out 70 × 80 mm CFRP panels made of 10 prepreg plies, with all the interfaces nano-modified (Figure S3). To ensure CFRP homogeneity, edge parts of panel (≈ 15 mm) were discarded.

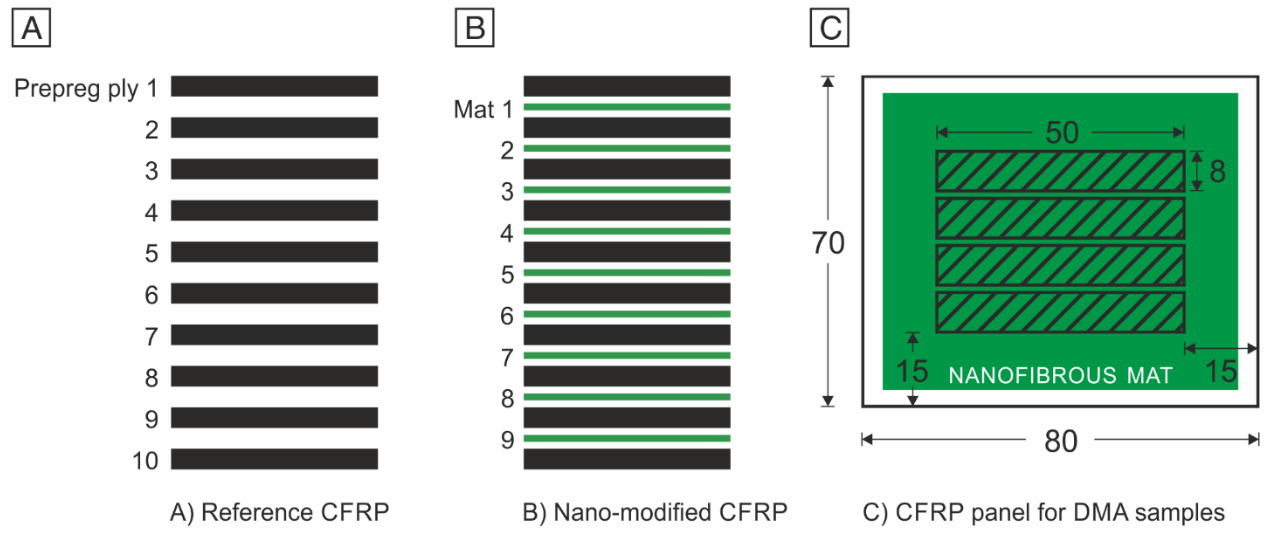

Figure S3. Stacking sequence of plies/mats for DMA panels production: (A) unmodified and (B) nano-modified CFRP laminates. (C) Dimensions (in mm) of CFRP panels and specimens for DMA.

S3 – Evaluation of the solubility parameters for NBR and Nomex and their miscibility

The solubility parameter δ, or Hildebrand solubility parameter, can be divided into three different components, the Hansen solubility parameters, according to Equation S1

$$\delta^2 = \delta_{\mathrm{d}}^2 + \delta_{\mathrm{p}}^2 + \delta_{\mathrm{h}}^2 \quad \text{Equation S1}$$

where δ_d is the factor accounting for dispersive forces, δ_p relates to polar forces and δ_h accounts for hydrogen bonding ability.[1] The Hansen solubility parameters can be evaluated according to the group contribution theory, summing up the contributions of each group composing the chemical structure of the material, in agreement with the following formulas, Equations S2-S4

$$\delta_{\mathrm{d}} = \frac{\sum_i F_{\mathrm{d}i}}{V} \quad \text{Equation S2}$$

$$\delta_{\mathrm{p}} = \frac{\sqrt{\sum_i F_{\mathrm{p}i}^2}}{V} \quad \text{Equation S3}$$

$$\delta_{\mathrm{h}} = \sqrt{\frac{\sum_i E_{\mathrm{h}i}}{V}} \quad \text{Equation S4}$$

using values reported in the literature typical for each group's $F_{\mathrm{d}i}$, $F_{\mathrm{p}i}$, $E_{\mathrm{h}i}$ and V [1,2]

One of the most common approaches, the one proposed by Hoftyzer and van Krevelen,[3] states that each component (δ_{d}, δ_{p} and δ_{h}) can be evaluated for each polymer according to the groups' contribution theory, summing up the contributions of each group composing the compound and using values reported in the literature for the group's $F_{\mathrm{d}i}$, $F_{\mathrm{p}i}$, $E_{\mathrm{h}i}$ and V. [1,2]

While the evaluation of such parameter for copolymers might not be exact, nonetheless the values obtained in Table S3 for NBR and Nomex might help to define the relative miscibility of the two polymers, according to the following Equation S5

$$\overline{\Delta\delta} = \sqrt{(\delta_{\mathrm{d}PCL} - \delta_{\mathrm{d}NBR})^2 + \left(\delta_{\mathrm{p}PCL} - \delta_{\mathrm{p}NBR}\right)^2 + (\delta_{\mathrm{h}PCL} - \delta_{\mathrm{h}NBR})^2} \quad \text{Equation S5}$$

A good miscibility occurs when $\overline{\Delta\delta}$ value is small, i.e. $\overline{\Delta\delta} < 5\text{MPa}^{1/2}$, and partial miscibility could potentially be attained up to $10\text{MPa}^{1/2}$.[2,4] Evaluation of the Hansen solubility parameters for PCL was easily found in the literature,[5] while a correct assessment of the solubility parameters for the actual carboxylated NBR polymer composition used in the present work was not available. Since it has been reported that variation of few percentage in the monomer composition could strongly affect the solubility parameters,[6] such calculations were carried out for the specific polymer presently used, that is actually composed of three different repeating units derived from their related monomers, butadiene (Bu), acrylonitrile (ACN) and methacrylic acid (MAA).

For the present paper, the two polymers involved, NBR and PCL, can be represented according to the formulas reported in Scheme S1.

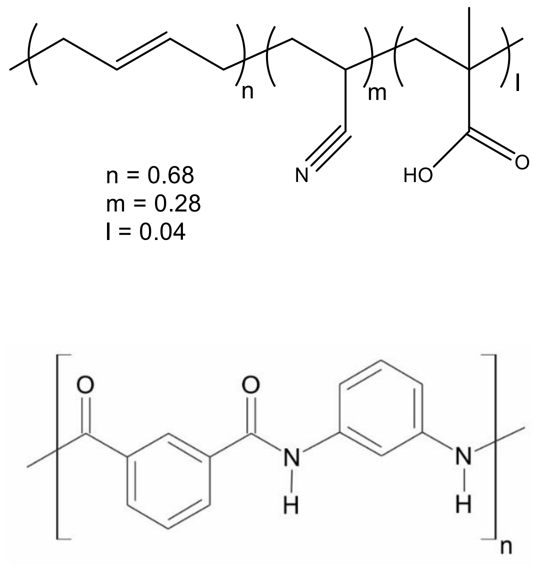

Scheme S1. NBR and Nomex.

In this case V, the molar volume of the structural unit of the polymer NBR, has been evaluated according to the following Equation S6

$V = \sum N_i V_i$ Equation S6

Table S2. Parameters for NBR and Nomex Hoftyzer and van Krevelen groups' contribution calculation [7]

Group		V_i (cm^3/mol)	F_{di} ($J^{1/2}$•$cm^{3/2}$•mol^{-1})	F_{pi} ($J^{1/2}$•$cm^{3/2}$•mol^{-1})	E_{hi} (J/mol)
**Butadiene (n=0.68)**					
$-CH_2-$	2x	15.55	270	0	0
=CH-	2x	13.18	200	0	0
**Acrylonitrile (m=0.28)**					
$-CH_2-$	1x	15.55	270	0	0
>CH-	1x	9.56	80	0	0
-CN	1x	23.1	430	1100	2500
**Methacrylic Acid (l=0.04)**					
$-CH_3$	1x	21.55	420	0	0
$-CH_2-$	1x	15.55	270	0	0
>C<	1x	3.56	-70	0	0
-COOH	1x	26.1	530	420	10000

<table>
<tr><th colspan="2">Group</th><th>V_i
(cm^3/mol)</th><th>F_{di}
($J^{1/2}$•$cm^{3/2}$•mol^{-1})</th><th>F_{pi}
($J^{1/2}$•$cm^{3/2}$•mol^{-1})</th><th>E_{hi}
(J/mol)</th></tr>
<tr><td colspan="6">PMIA</td></tr>
<tr><td>-C=O</td><td>2x</td><td rowspan="2">28.3</td><td>290</td><td>770</td><td>2000</td></tr>
<tr><td>-N-H</td><td>2x</td><td>160</td><td>210</td><td>3100</td></tr>
<tr><td>$-CH_{ar}$ (4x)</td><td rowspan="2">2x</td><td>13.42x4</td><td rowspan="2">1270</td><td rowspan="2">110</td><td rowspan="2">0</td></tr>
<tr><td>$-C_{ar}$ (2x)</td><td>7.42x2</td></tr>
</table>

Hereabove, in Table S2, are the values used for evaluating the Hansen solubility parameters for NBR and Nomex, according to the method of Hoftyzer and van Krevelen.[2]

The Hansen solubility parameters obtained according to Equations S2-S4, are finally reported in Table S3.

Table S3. Hansen solubility parameters used for evaluation of NBR and Nomex theoretical miscibility

	δ_d	δ_p	δ_h
**NBR**	13.2	10.6	4.5
**NOMEX**	17.8	8.3	7.2

Based on the solubility parameter evaluation in Table S3 and according to Equation S5, $\overline{\Delta\delta}$ value for the NBR/Nomex pair was found to be 5.7 $MPa^{1/2}$, a value that does not account for a complete miscibility of the two components, tending instead toward an immiscible system.

References

(1) Cipitria, A.; Skelton, A.; Dargaville, T. R.; Dalton, P. D.; Hutmacher, D. W. Design, Fabrication and Characterization of PCL Electrospun Scaffolds - A Review. *J. Mater. Chem.* **2011**, *21* (26), 9419–9453. https://doi.org/10.1039/c0jm04502k.

(2) Van Krevelen, D. W.; Te Nijenhuis, K. Cohesive Properties and Solubility. In *Properties of Polymers*; 2009; Vol. i, pp 189–227. https://doi.org/10.1016/b978-0-08-054819-7.00007-8.

(3) Van Krevelen, D. W.; Te Nijenhuis, K. Cohesive Properties and Solubility. In *Properties of Polymers*; Van Krevelen, D. W., Te Nijenhuis, K. B. T.-P. of P. (Fourth E., Eds.; Elsevier: Amsterdam, 2009; pp 189–227.

(4) Shaw, M. T. Preparation of Blends. In *Polymer Blends and Mixtures*; Springer Netherlands, 1985; pp 57–67.

(5) Chandler, L. A.; Collins, E. A. Multiple Glass Transitions in Butadiene-Acrylonitrile Copolymers. *Rubber Chem. Technol. 43*, 1465–1472.

(6) Ono, H.; Fujiwara, H.; Nishimura, S. Nanoscale Heterogeneous Structure of Polyacrylonitrile-Co-Butadiene with Different Molecular Mobilities Analyzed by Spin−Spin Relaxation Time. *Polym. J.* **2013**, *45*, 1027–1032.

(7) Barton, A. F. M. *Handbook of Solubility Parameters and Other Cohesion Parameters (2nd Edition)*; 1991.

S4 – Morphological characterization of nanofibrous mats

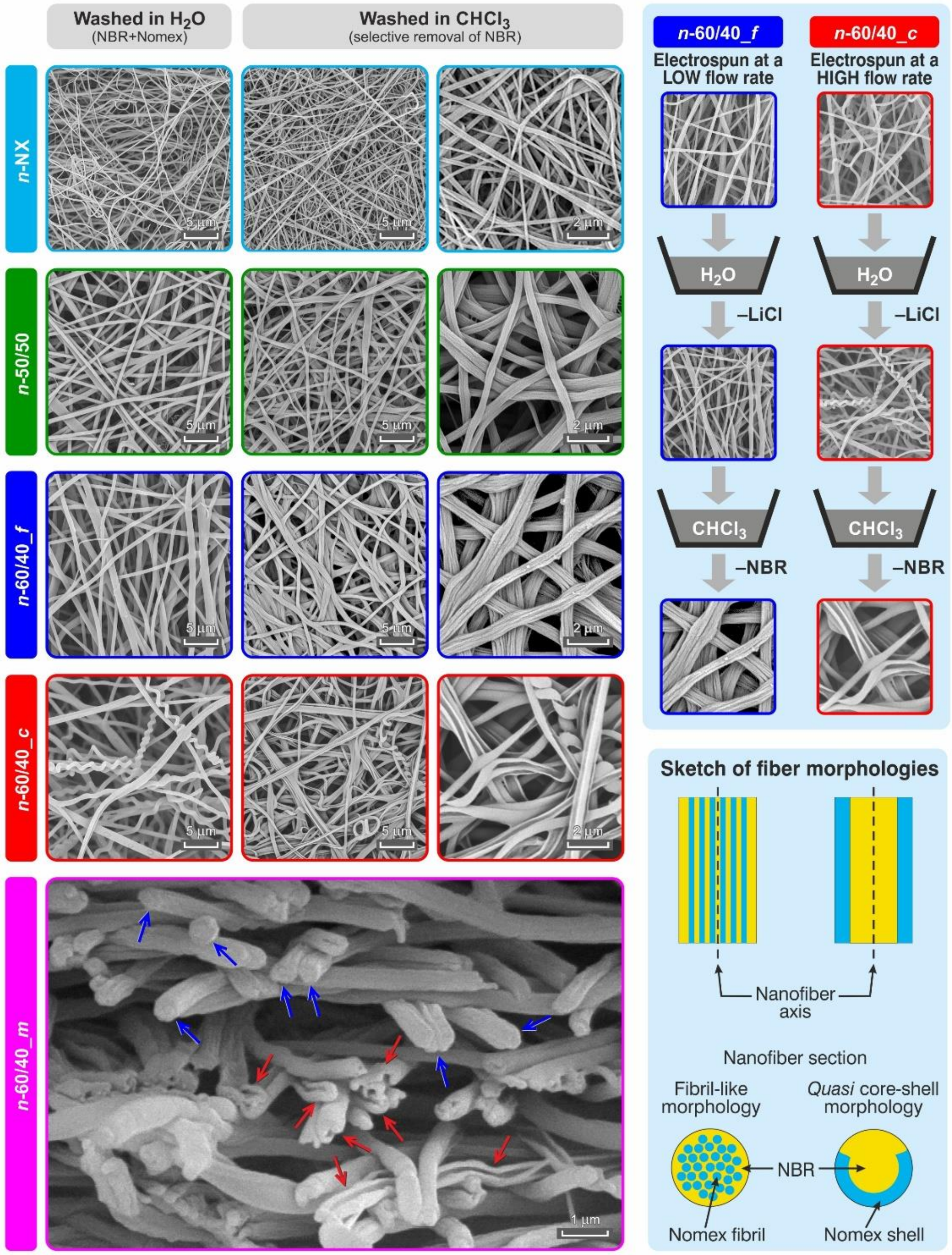

Figure S4. SEM micrographs of produced nanofibers after washing in water for LiCl removal (1st column) and after washing in chloroform to eliminate NBR (2nd and 3rd columns). For *n*-60/40_*m* it is reported only the image (section view) after NBR removal (blue arrows: *n*-60/40_*f* nanofiber type, red arrows: *n*-60/40_*c* nanofiber type). On the right, it is reported the washing procedure for NBR selective removal and a sketch of the two very different nanofiber morphologies due to different electrospinning conditions (NBR in yellow, Nomex in cyan).

S5 – NBR/Nomex emulsion: optical images and ATR-IR of the different phases

The opaque aspect of NBR/Nomex blends strongly suggests emulsion formation, confirmed by optical micrographs taken at different times of mixing of NBR and Nomex in $CHCl_3$/DMAc solvent system (Figure S5).

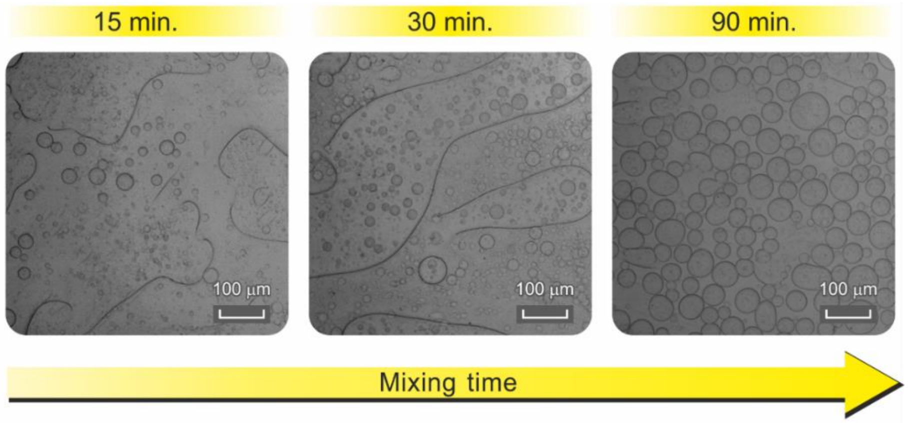

Figure S5. Optical images of *s*-60/40 in $CHCl_3$/DMAc solvent system showing NBR/Nomex emulsion after 15, 30 and 90 minutes the mixing of NBR and Nomex solutions under magnetic stirring.

To better understand the behaviour and composition of the NBR/Nomex emulsion, 20 mL of *s*-60/40 mixture (60% NBR with respect to the polymeric fraction) was poured into a cylinder and left to rest (Figure S6A). After 12 hours, the emulsion shows 3 phases (Figure S6B). Over time, the proportion of phases changes, with an increase of the bottom one (Figure S6C,D), until forming two phases after 4 days (Figure S6E).

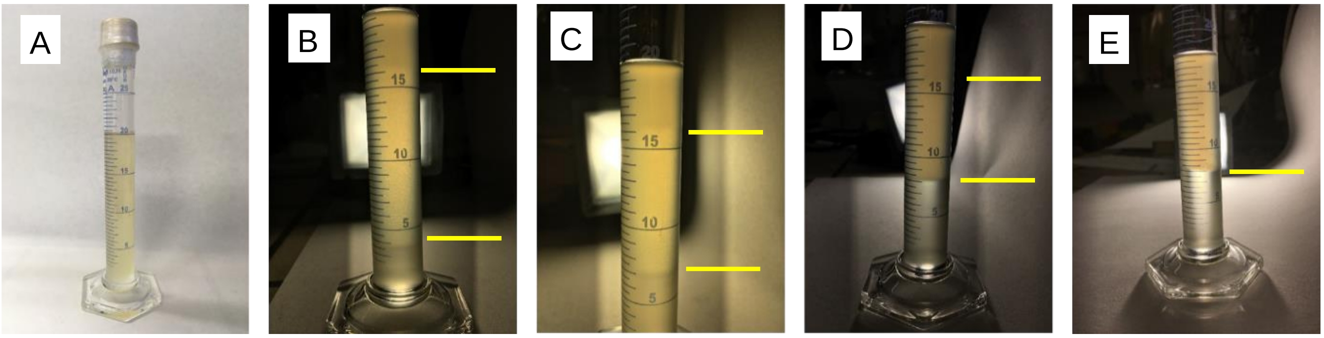

Figure S6. Emulsion stability test conducted on *s*-60/40 mixture: emulsion aspect at the beginning (A), after 12 (B), 24 (C), 48 (D), and 96 (E) hours.

In Figure S7 are reported the IR spectra of NBR (dry fraction from *s*-NBR_*b*), Nomex (dry fraction from *s*-NBR_*b*) and of the three phases formed after 12 hours (Figure S6B). The bottom phase, which appears clear, is constituted by Nomex only. The central one is constituted by both NBR and Nomex, as well as the top phase.

The IR spectra on the two phases after 96 hours (Figure S6E) reveals that the bottom phase is constituted by only Nomex, while in the top phase both NBR and Nomex are present.

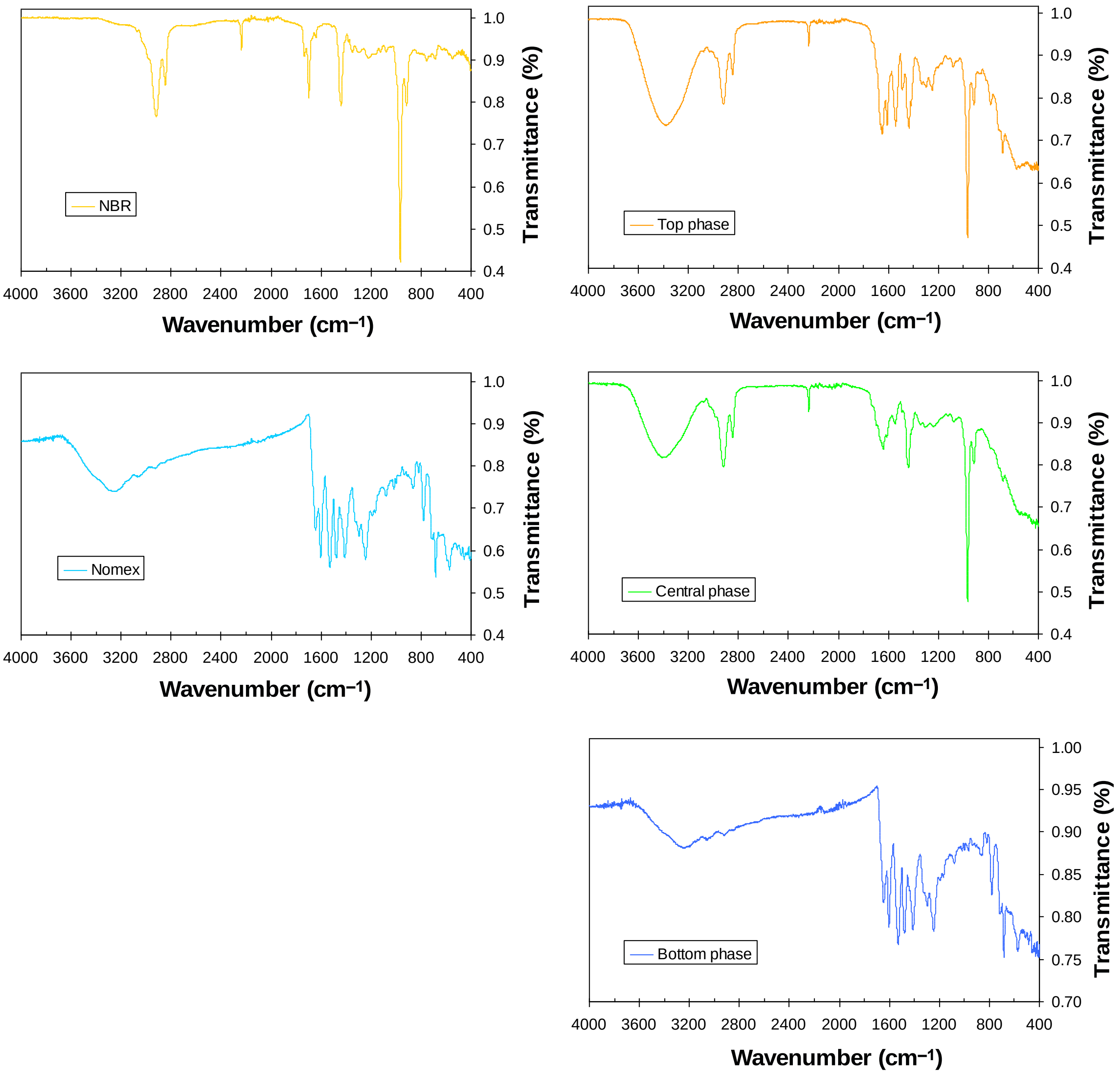

Figure S7. ATR-IR spectra of NBR, Nomex, and of the three phases visible in Figure S6B (bottom, central and top phases).

S6 – Data fitting of stress-strain tensile testing curves

The data fitting of the stress-strain curves of nanofibrous mats was obtained by applying the following equation (Equation 2 in the main text):

$$\sigma(\varepsilon) = a\varepsilon + b(1 - e^{-c\varepsilon})$$

where a, b, c are parameters used to attain the data fitting.

The data fitting was performed using the Solver tool implemented in Microsoft Excel, by minimizing the sum of the square errors (method of least squares).

In Figure S8 are reported selected exempla of the application of the data fitting model. Each curve is representative of each membrane type.

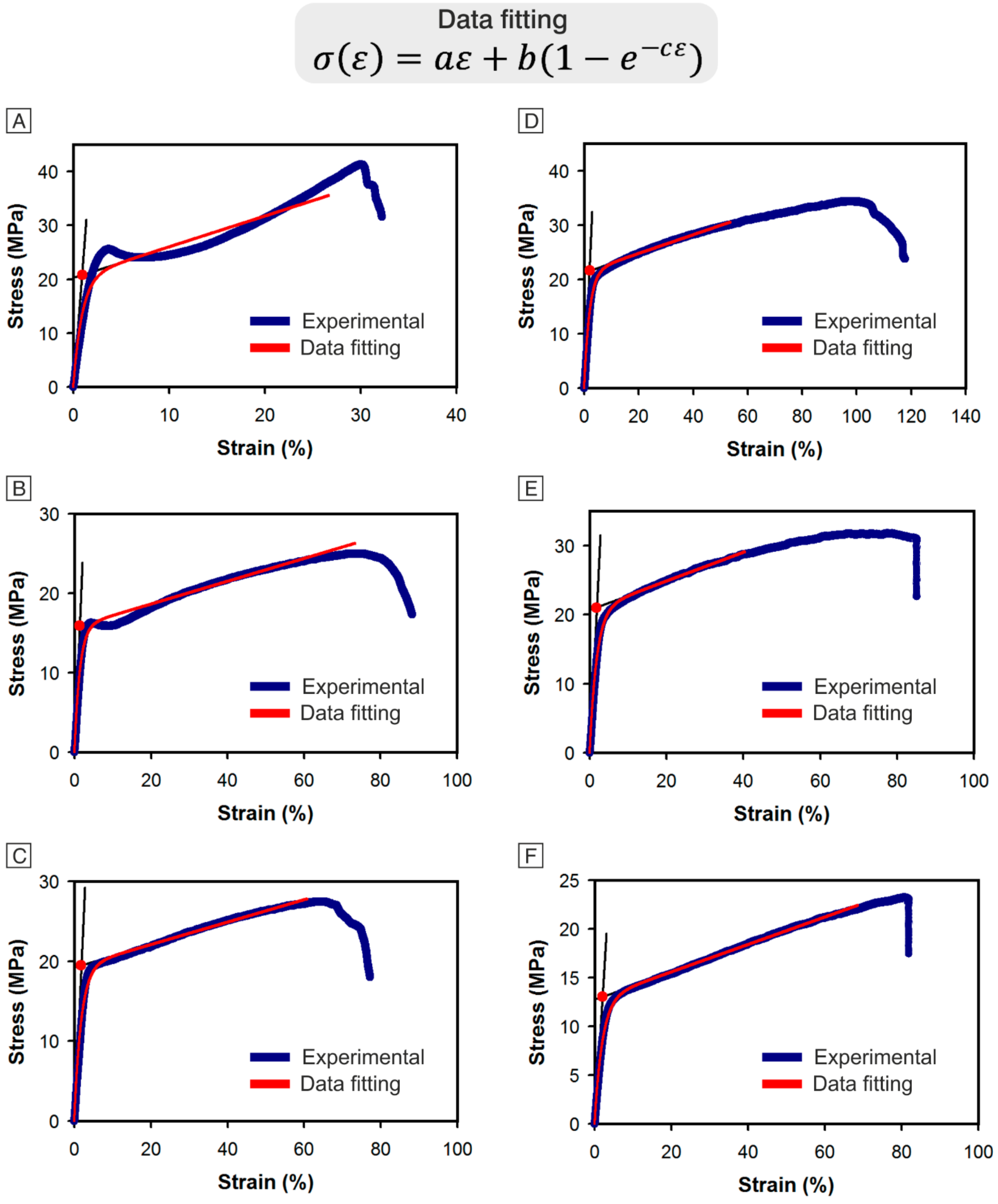

Figure S8. Application of the data fitting model on tensile stress-strain curves of nanofibrous mats: A) *n*-NX, B) *n*-40/60, C) *n*-50/50, D) *n*-60/40_*f*, E) *n*-60/40_*m*, F) *n*-60/40_*c*.

S7 –Mode I and Mode II interlaminar fracture toughness of nano-modified CFRPs

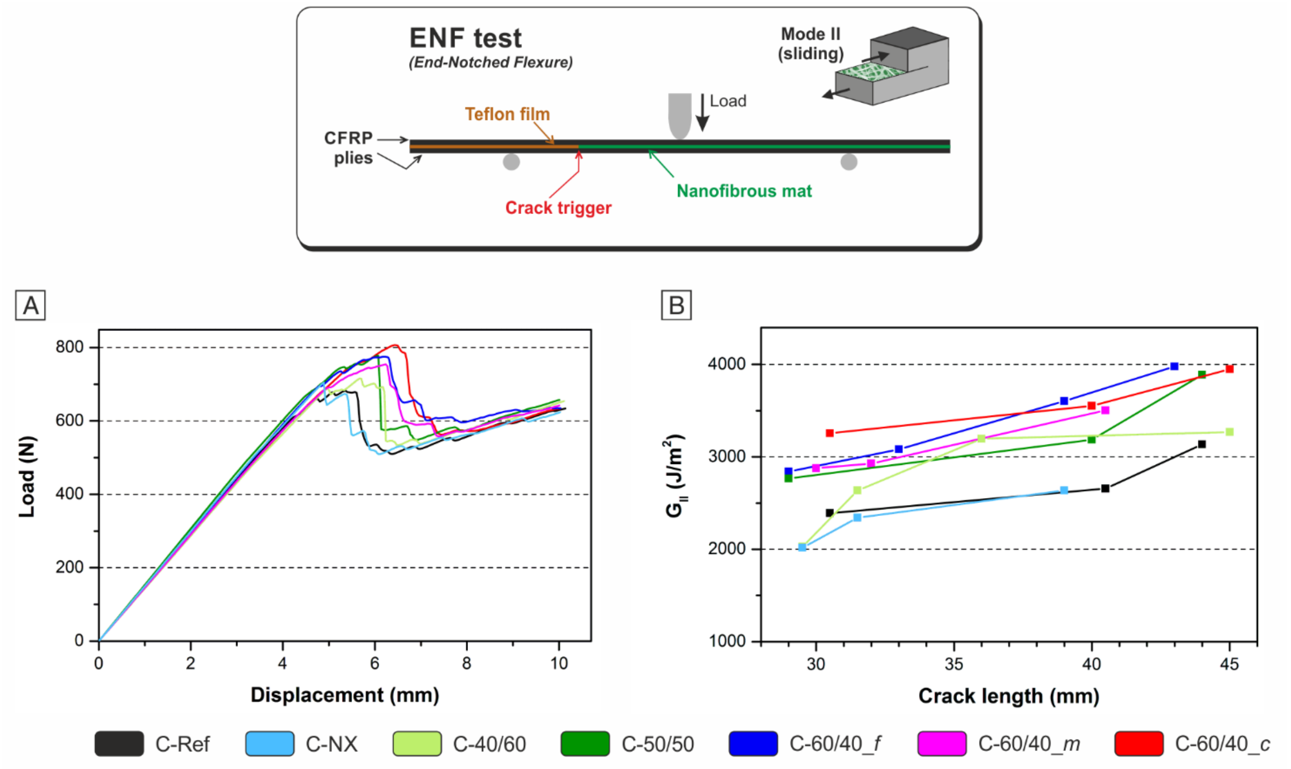

Figure S9. ENF tests results: A) load-displacement curves; B) *R*-curves related to the same specimens displayed in A).

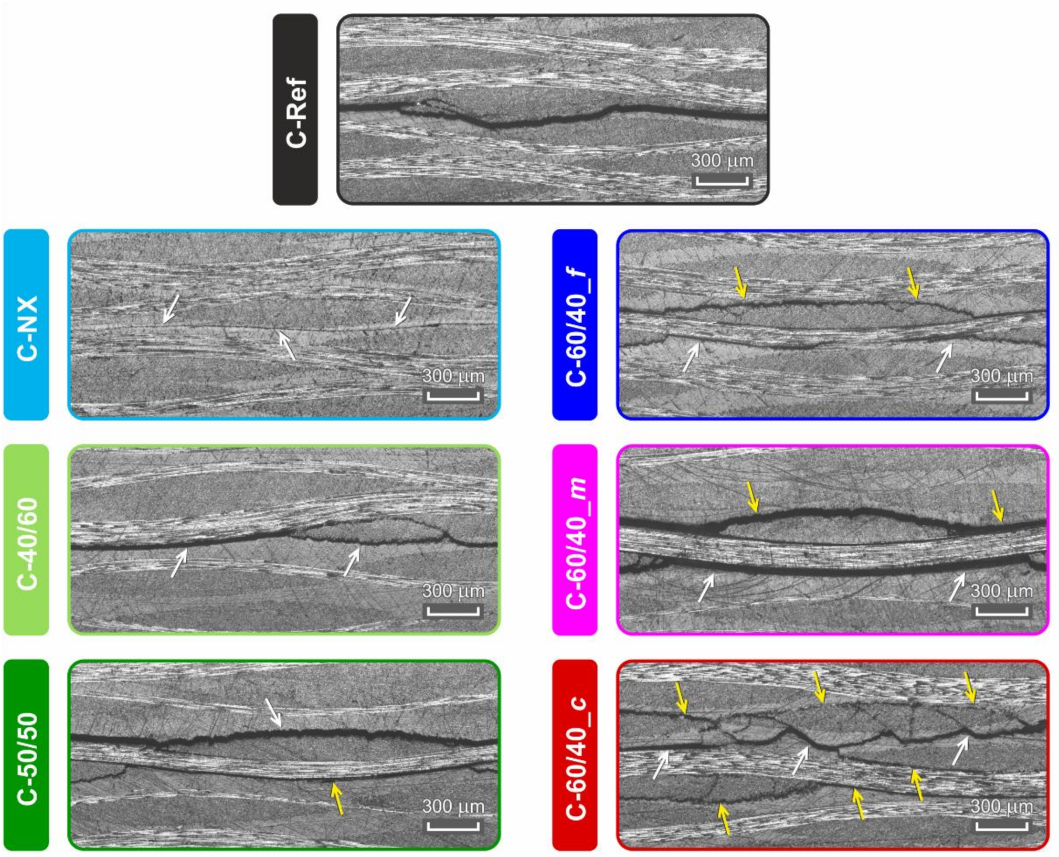

Figure S10. Micrographs of DCB specimens after the delamination tests of nano-modified CFRPs, besides the reference laminate. White arrows: designed crack plane (central plane); yellow arrows: plane(s) adjacent to the central plane.

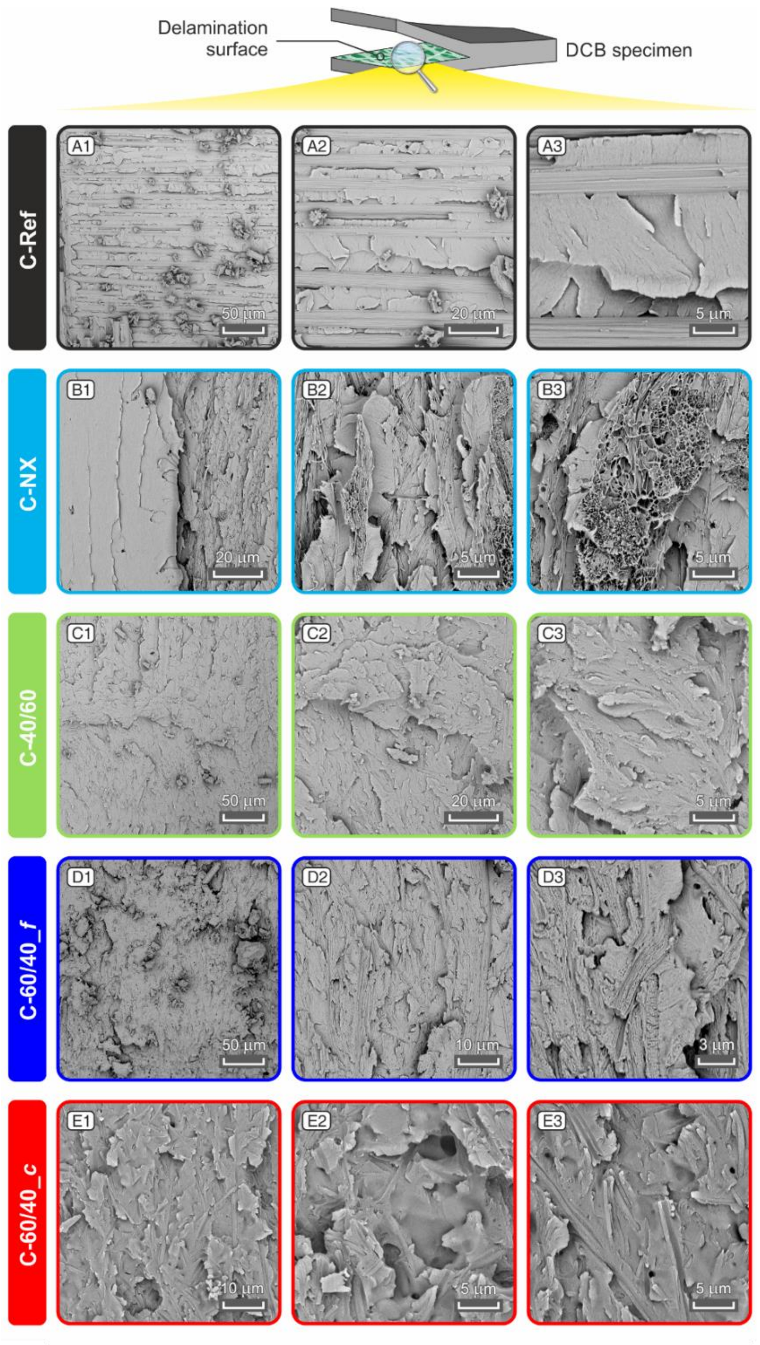

Figure S11. Micrographs of DCB specimens after the delamination tests: morphology of delamination surfaces. 1st column (from A1 to E1): general view; 2nd and 3rd columns (from A2 to E2 and from A3 to E3, respectively): details of matrix morphology and nanofibrous mat (where present).

Table S4. Thermomechanical properties of CFRP laminates tested via DMA.

CFRP	Interleaved mat	E' @ 25 °C	E' onset	T_α
		GPa	°C	°C
C-Ref	—	39.7 ± 1.2	122 ± 1	153
C-NX	*n*-NX	35.2 ± 1.1	115 ± 1	148
C-40/60	*n*-40/60	38.5 ± 1.2	121 ± 1	152
C-50/50	*n*-50/50	37.3 ± 1.3	120 ± 1	151
C-60/40_*c*	*n*-60/40_*c*	37.5 ± 1.0	118 ± 2	149
C-60/40_*m*	*n*-60/40_*m*	36.8 ± 1.3	118 ± 2	149
C-60/40_*f*	*n*-60/40_*f*	37.1 ± 1.2	118 ± 1	150
